# First-principles calculations of equilibrium Ga isotope fractionations between several important Ga-bearing minerals and aqueous solutions

**DOI:** 10.1038/s41598-023-32858-0

**Published:** 2023-04-17

**Authors:** Jixi Zhang

**Affiliations:** School of Geography and Environmental Science (School of Karst Science), Guizhou Normal University/State Engineering Technology Institute for Karst Desertification, Guiyang, 550001 China

**Keywords:** Geochemistry, Geochemistry

## Abstract

This study predicts the equilibrium isotope fractionation factors for some important Ga-bearing species, including major minerals, aqueous solutions and gas phase systems. Equilibrium isotope fractionations of Ga were investigated by using the first-principles quantum chemistry method at the B3LYP/6-311+G(d) level. The 10^3^ln(RPFR) values of orthoclase, albite, quartz, kaolinite, forsterite, montmorillonite, gibbsite, cassiterite, aragonite, sphalerite and calcite were calculated with the volume variable cluster model. The 10^3^ln(RPFR)s of these minerals decrease in the following order: orthoclase > albite > quartz > kaolinite > forsterite > montmorillonite > gibbsite > cassiterite > aragonite > sphalerite > calcite. The solvation effect of Ga^3+^-bearing aqueous species is modeled by the water-droplet method, and the 10^3^ln(RPFR)s of Ga^3+^-bearing aqueous species decrease in the following order: [Ga(OH)_4_]^−^ > [Ga(OH)_3_] > [Ga(OH)]^2+^ > [Ga(OH)_2_]^+^ > [Ga(H_2_O)_6_]^3+^. The calculation results show that equilibrium isotope fractionations of Ga between different minerals, solutions and gas phases are appreciable. Among minerals, Ga isotope fractionation exhibits the largest value between orthoclase and calcite. Ga isotopic fractionation factor between these two minerals can reach 3.18 per mil at 100 °C. Ga isotope fractionations between Ga-bearing aqueous species and minerals are important for obtaining information about the different geochemical processes, such as surficial geochemistry. This study has provided important Ga isotope fractionation factors.

## Introduction

Gallium (Ga) is among the most important trace elements in the crust and mantle and hardly forms an independent mineral^1^. The abundance of Ga in the Earth's crust is approximately 15–19 ppm^2^. Ga-Al is a geochemically related pair of elements that could coexist during various geochemical processes, such as crustal evolution or weathering^3,4^. Because Ga and Zn are adjacent elements belonging to the same period, they exhibit some similar chemical properties, such as ion radii and chemical properties. Ga could enter Al-bearing and Zn-bearing mineral crystal lattices in the form of isomorphism^5^. Hence, the common Ga-bearing minerals are bauxite, cassiterite and sphalerite.

Ga has two stable isotopes, i.e., ^69^Ga and ^71^Ga with abundances of 60.11% and 39.89%, respectively^6,7^. The mass difference between the two isotopes (^69^Ga and ^71^Ga) can reach 2.9%, and Ga isotope fractionation can occur in different processes^1^. The latest research on Ga isotope fractionation shows that Ga isotope fractionation in natural samples is obvious. Thus, Ga isotopes can be used as an emerging geochemical tool to study a range of geochemical processes, including both low-temperature weathering and high-temperature evaporation^1,8–14^.

Although the general rule of isotopic equilibrium fractionation is that equilibrium fractionation becomes very small at high temperatures, studies on weathering processes, adsorption processes, and Ga isotopic fractionation of carbonaceous chondrites have revealed that Ga isotopic fractionation is relatively significant. Previous studies have found that the Ga isotope fractionations of terrestrial samples are very small^11–14^. Ga isotope fractionation can occur in many processes, such as evaporation and adsorption of mineral particles^10^. These results indicated that Ga isotope composition variations commonly exist in natural samples. In the past few years, Ga isotopes have been increasingly used in the field of early Earth evolution and the environment. Ga isotope fractionation data are represented by the symbol δ.

$$\delta {}^{71}Ga = \left[ {\frac{{\left( {{{{}^{71}Ga} \mathord{\left/ {\vphantom {{{}^{71}Ga} {{}^{69}Ga}}} \right. \kern-0pt} {{}^{69}Ga}}} \right)}}{{\left( {{{{}^{71}Ga} \mathord{\left/ {\vphantom {{{}^{71}Ga} {{}^{69}Ga}}} \right. \kern-0pt} {{}^{69}Ga}}} \right)_{Ga - IPGP - s\tan dard} }} - 1} \right] \times 1000$$

With the development of multiple collector inductively coupled plasma‒mass spectrometry (MC-ICP‒MS), isotope analysis precision has increased substantially, which enables us to accurately obtain Ga isotope compositions of various samples. Ga is a typical moderately volatile element with a half-mass condensation temperature Tc (at 10^–4^ bar) 968K^6^. Kato and Moynier (2017) revealed that calcium–aluminum-rich inclusions (CAIs) are enriched in Ga compared to the bulk meteorite, and the isotope fractionation value could reach up to − 3.56‰ (δ^71^Ga)^12^. Thus, large Ga isotope fractionation still occurs between CAIs and solid meteorites at high temperatures. The study of Ga isotope fractionation provides another way to investigate the details of evaporation processes. Volatile elements play a key role in the chemical evolution of planets. Previous studies have shown that the Moon is volatile-depleted and that stable isotope fractionations of moderately volatile elements could occur during the giant-impact event of Moon formation^15–17^. Ga isotopes could be fractionated in vaporization processes of the Moon but hardly fractionated during igneous processes of the Earth within analytical uncertainty^12^. Therefore, Ga is a very important element to investigate the evaporation processes due to the giant impact.

The main difficulty in constructing the theory of Ga isotope geochemistry and better understanding the mechanism of Ga isotope fractionation is the lack of quantitative isotopic fractionation data between minerals and between minerals and solutions as a function of temperature. The experimental results demonstrate that Ga isotope fractionation in chemical weathering, adsorption and gasification processes is a kind of kinetic fractionation^1,8,10–14,18^. Until now, Ga isotope equilibrium fractionation data have not been accurately obtained. This greatly limits the application of Ga isotopes in a series of geochemical processes.

The rapid development of computational geochemistry (first principles, etc.) provides an excellent opportunity to accurately obtain these theoretical parameters. To date, many theoretical calculations about traditional and nontraditional isotope systems have been performed, but there is no theoretical calculation of Ga isotopes between minerals and between minerals and solutions^19–27^. Furthermore, isotopic fractionation data for Ga-bearing gaseous species are not available, which may limit the use of Ga isotopes as a geochemical tool.

In this research, the quantum chemistry method was adopted to obtain Ga isotope fractionation factors between different Ga-bearing substances. Ga_2_O, Ga_2_O_3_, Ga_2_S_3_, GaCl_3_, GaF_3_ and GaO in gaseous phases; [Ga(H_2_O)]^3+^, [Ga(OH)]^2+^, [Ga(OH)_2_]^+^, [Ga(OH)_3_] and [Ga(OH)_4_]^−^ in aqueous phases; and a series of minerals, such as montmorillonite, albite, aragonite, calcite, cassiterite, diaspore, forsterite, gibbsite, kaolinite, orthoclase, quartz and sphalerite, in solid phases were chosen as important geological Ga isotope systems for study. The Ga isotope fractionation of the aqueous solution environment and evaporation process were studied in this study. These factors are expected to provide important basic data for researchers who investigate Ga isotope fractionation in several geoscience subdisciplines.

## Results

### Structural parameters of the Ga-bearing minerals, solutions and gas phases

**Figure 1:**
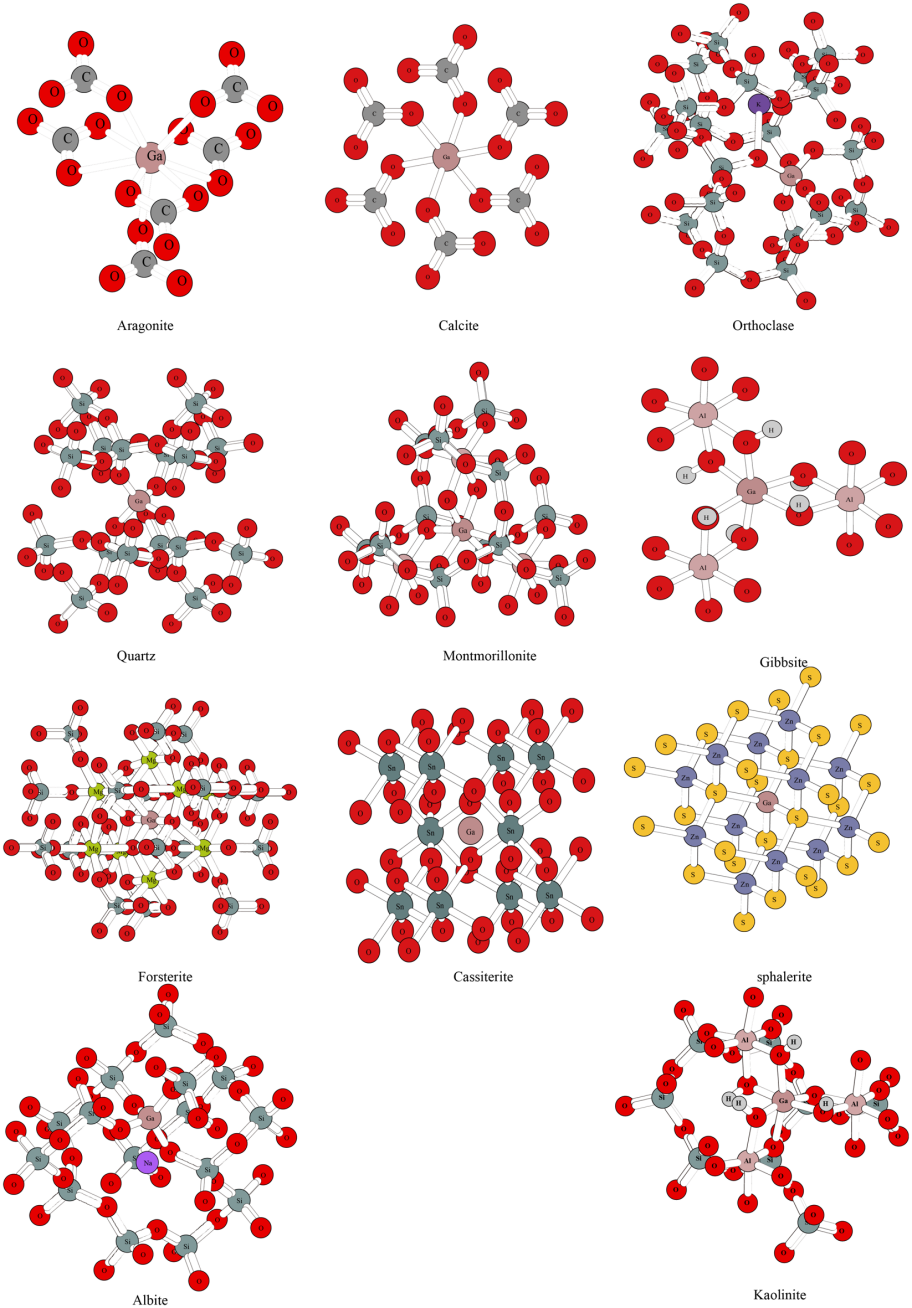
The cluster structures of orthoclase, albite, quartz, kaolinite, forsterite, montmorillonite, gibbsite, cassiterite, aragonite, sphalerite and calcite with Ga atoms in center. The coordination number of central Ga atoms for aragonite is nine, for kaolinite, forsterite, montmorillonite, gibbsite, cassiterite and calcite are six, for albite, orthoclase, sphalerite and quartz are four.

The cluster structures of orthoclase, albite, quartz, kaolinite, forsterite, montmorillonite, gibbsite, cassiterite, aragonite, sphalerite and calcite are shown in Fig. 1. Ga is fourfold coordinated in albite, orthoclase, sphalerite and quartz, sixfold coordinated in kaolinite, forsterite, montmorillonite, gibbsite, cassiterite and calcite, but ninefold coordinated in aragonite. The calculated cluster structure parameters are presented in Table S1 in the supplementary file. For forsterite contains different caution sites. To evaluate their contributions to the total RPFR value, the structural optimization and frequency calculation for both structures were carried out, and the RPFR factors were also calculated. The structures of forsterite with different caution sites are given in Fig. S1, named M1 and M2. The method of isomorphism was used to obtain the structures of the Ga-bearing minerals mentioned above. Data on the crystal structures of these minerals (Ga substitution) were not available, and therefore, no data comparison was made in this article. The VVCM method is a kind of cluster method, and the calculated frequencies of these Ga-bearing minerals cannot be compared with the experimental frequencies.

**Figure 2:**
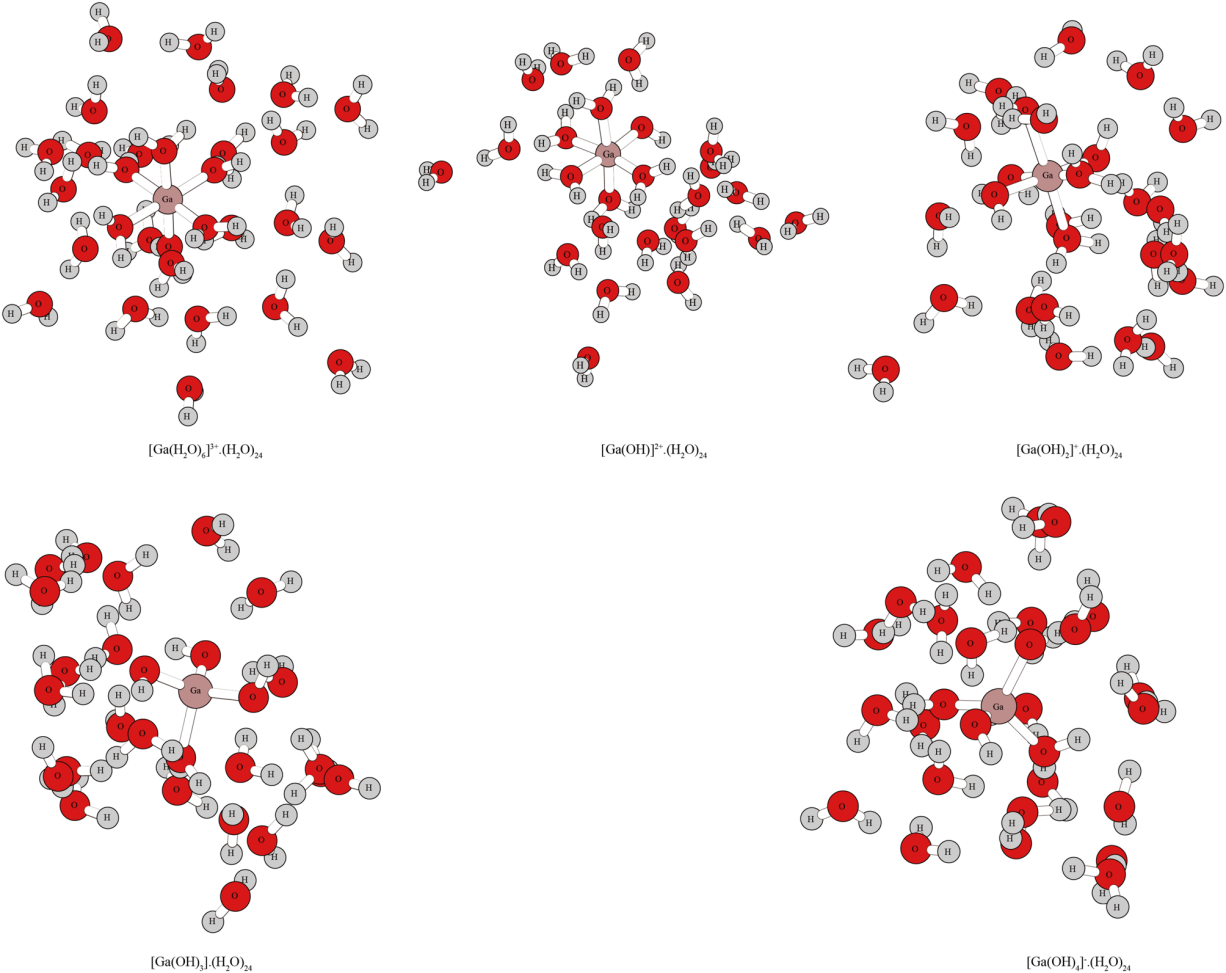
The cluster structures of Ga-bearing solutions. The spatial structures for [Ga(H_2_O)_6_]^3+^·(H_2_O)_24_, [Ga(OH)]^2+^·(H_2_O)_24_ and [Ga(OH)_2_]^+^·(H_2_O)_24_, [Ga(OH)_3_]·(H_2_O)_24_ and [Ga(OH)_4_]^−^.(H_2_O)_24_ are octahedron and tetrahedron, respectively. The number of bonds around the Ga^3+^ represents the number of ligands for Ga^3+^.

The cluster structures of Ga-bearing solutions ([Ga(H_2_O)_6_]^3+^·(H_2_O)_24_, [Ga(OH)_2_]^+^ (H_2_O)_24_, [Ga(OH)]^2+^ (H_2_O)_24_, [Ga(OH)_3_] (H_2_O)_24_ and [Ga(OH)_4_]^−^ (H_2_O)_24_) are shown in Fig. 2. The optimization results show that Ga is fourfold coordinated in [Ga(OH)_3_] (H_2_O)_24_ and [Ga(OH)_4_]^−^ (H_2_O)_24_ but sixfold coordinated in [Ga(H_2_O)_6_]^3+^ (H_2_O)_24_, [Ga(OH)_2_]^+^ (H_2_O)_24_ and [Ga(OH)]^2+^ (H_2_O)_24_. The calculated results are in agreement with the previous experimental results^28^. The calculated cluster structure parameters are presented in Table S2 in the supplementary file. The first-coordination shells of these species are shown in Fig. S2.

**Figure 3:**
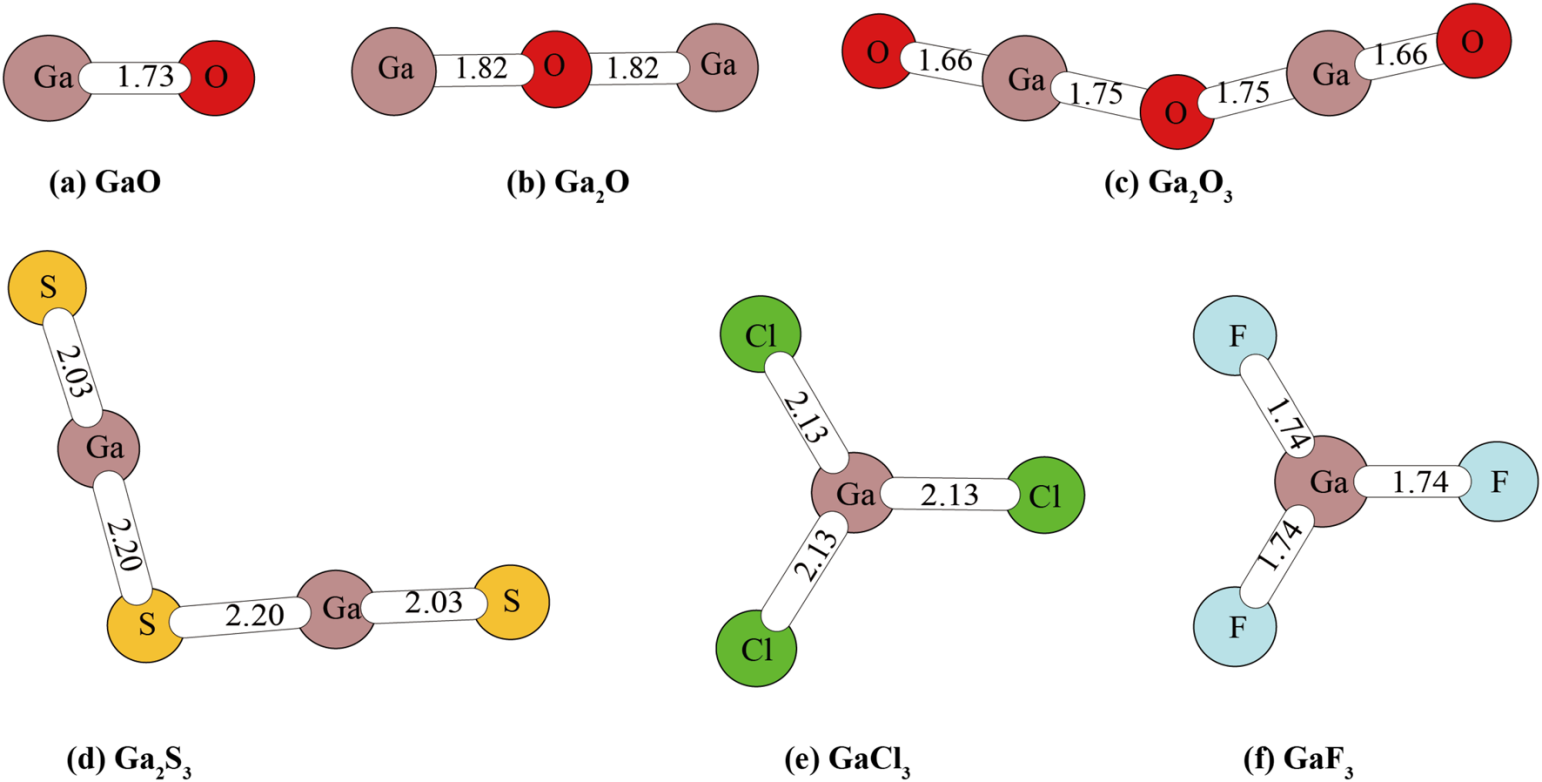
The structures of Ga-bearing gas phases. The numbers in this figure stand for bond lengths of different Ga-N bonds which N represents atoms O, S, Cl and F.

The structures of the Ga-bearing gas phases** (**Ga_2_O, Ga_2_O_3_, Ga_2_S_3_, GaCl_3_, GaF_3_ and GaO) are shown in Fig. 3. The calculated bond lengths for GaCl_3_ and GaF_3_ are 2.13 and 1.74 Å, agreeing well with the experimental values (GaCl_3_: 2.11, GaF_3_:1.73)^29^.

### Calculated Ga isotope fractionation data

#### The 10^3^ln(RPFR)s of different Ga-bearing aqueous species

**Figure 4:**
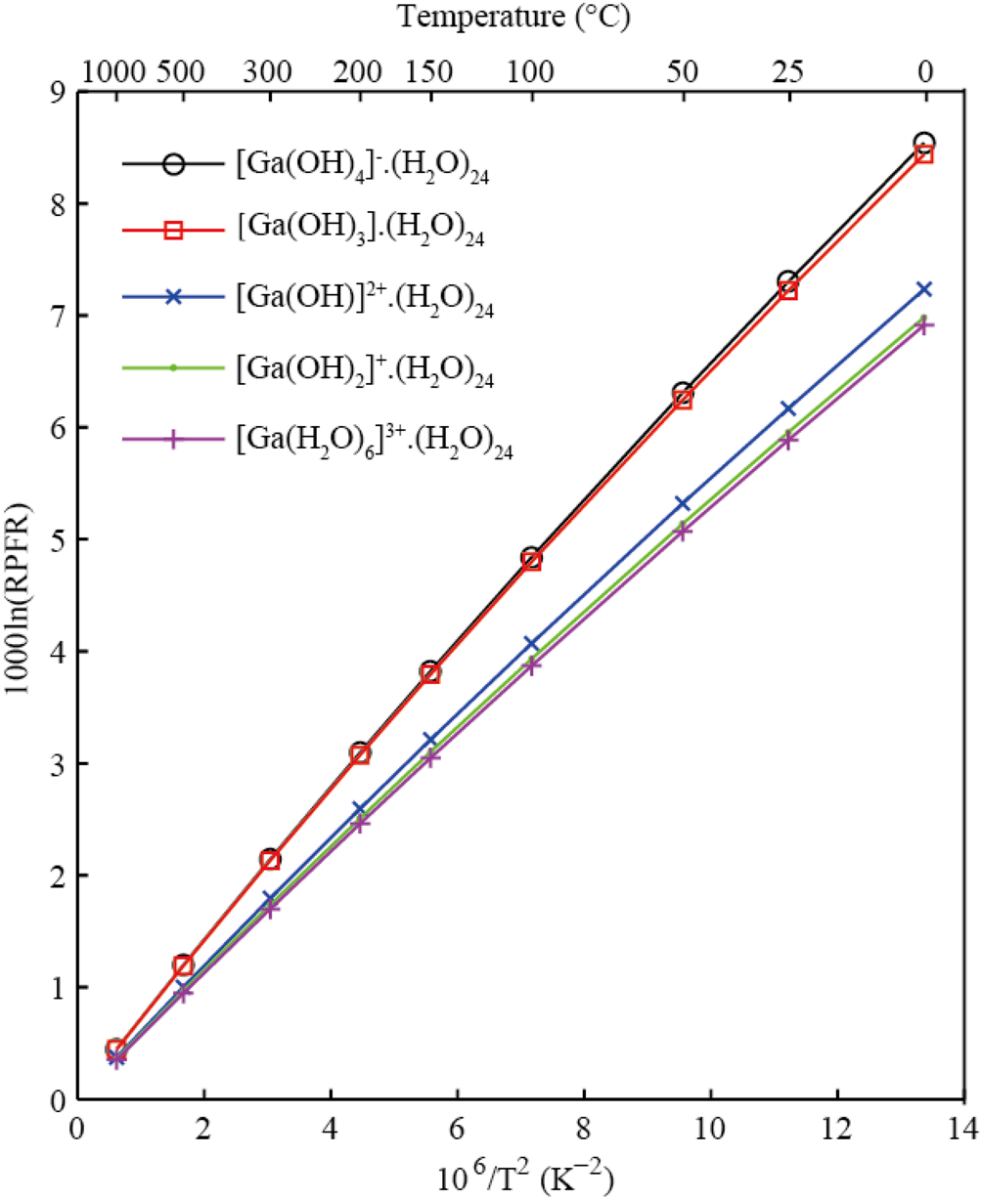
The 10^3^ln(RPFR)s of different Ga-bearing aqueous solutions as a function of temperature.

This study gives the mean values of four parallel calculations for five Ga-bearing aqueous solutions. The RPFR values decreased with increasing temperature for all species. The 10^3^lnRPFR values of Ga-bearing solutions at different temperatures calculated in this study are shown in Table 1. The polynomial expansions of the RPFR factors (^71^ Ga/^69^ Ga) of different Ga-bearing solutions based on the function 10^3^lnRPFR = ax + bx^2^ + cx^3^, with x = 10^6^/T^2^ (T is the temperature in Kelvin), are presented in Table 2. The 10^3^lnRPFR values of different Ga-bearing aqueous solutions as a function of temperature are given in Fig. 4. For example, 10^3^ln(RPFR) values of these five species at 25℃ range from 5.89‰ to 7.30‰. Scaling factors were not applied when calculating the results in this study.

**Table 1:** 10^3^ln(RPFR) values of different Ga-bearing species at different temperatures.

	Temperature (℃)								
	0	25	50	100	150	200	300	500	1000
Minerals									
Orthoclase	9.60	8.21	7.09	5.44	4.30	3.48	2.41	1.35	0.50
Albite	9.30	7.95	6.87	5.27	4.16	3.37	2.33	1.30	0.49
Quartz	9.10	7.77	6.71	5.14	4.06	3.29	2.27	1.27	0.47
Kaolinite	6.51	5.53	4.76	3.62	2.85	2.30	1.58	0.88	0.33
Forsterite	6.05	5.14	4.41	3.35	2.63	2.12	1.45	0.81	0.30
Montmorillonite	5.70	4.84	4.15	3.16	2.48	2.00	1.37	0.76	0.28
Gibbsite	5.60	4.77	4.10	3.13	2.46	1.99	1.37	0.76	0.28
Cassiterite	4.97	4.20	3.59	2.72	2.12	1.71	1.17	0.65	0.24
Aragonite	4.41	3.74	3.22	2.45	1.93	1.55	1.07	0.60	0.22
Sphalerite	4.18	3.53	3.02	2.28	1.78	1.43	0.98	0.54	0.20
Calcite	4.12	3.48	2.99	2.26	1.77	1.43	0.98	0.54	0.20
Gas phases									
Ga_2_O	2.15	1.83	1.58	1.21	0.95	0.77	0.53	0.30	0.11
Ga_2_O_3_	7.11	6.16	5.38	4.20	3.37	2.75	1.93	1.10	0.42
Ga_2_S_3_	4.57	3.90	3.37	2.58	2.03	1.64	1.13	0.63	0.24
GaCl_3_	5.72	4.86	4.17	3.17	2.49	2.00	1.38	0.76	0.28
GaF_3_	9.05	7.79	6.77	5.24	4.16	3.38	2.36	1.32	0.50
GaO	2.48	2.13	1.85	1.44	1.14	0.93	0.65	0.36	0.14
Ga-solutions									
[Ga(OH)_4_]^−^·(H_2_O)_24_	8.54	7.30	6.31	4.84	3.82	3.10	2.14	1.20	0.45
[Ga(H2O)_6_]^3+^·(H_2_O)_24_	6.91	5.89	5.07	3.87	3.05	2.46	1.70	0.95	0.35
[Ga(OH)]^2+^·(H_2_O)_24_	7.23	6.17	5.32	4.07	3.21	2.60	1.79	1.00	0.37
[Ga(OH)_3_]·(H_2_O)_24_	8.44	7.22	6.25	4.80	3.79	3.07	2.13	1.19	0.45
[Ga(OH)_2_]^+^·(H_2_O)_24_	6.98	5.96	5.14	3.93	3.10	2.51	1.74	0.97	0.36

**Table 2:** Polynomial expansions of the RPFR factors (^71^ Ga/^69^ Ga) of different Ga-bearing species based on the function 10^3^lnβ = ax + bx^2^ + cx^3^, with x = 10^6^/T^2^ (T is the temperature in Kelvin).

	Coefficient		
	a	b	c
Solutions			
[Ga(OH)_4_]^−^·(H_2_O)_24_	0.7275	− 8.3137 × 10^–3^	1.1734 × 10^–4^
[Ga(H_2_O)_6_]^3+^·(H_2_O)_24_	0.5734	− 5.4087 × 10^–3^	8.2297 × 10^–5^
[Ga(OH)]^2+^·(H_2_O)_24_	0.6071	− 6.3203 × 10^–3^	9.6375 × 10^–5^
[Ga(OH)_3_] ·(H_2_O)_24_	0.7248	− 8.8660 × 10^–3^	1.3149 × 10^–4^
[Ga(OH)_2_]^+^·(H_2_O)_24_	0.5876	− 6.2963 × 10^–3^	9.7843 × 10^–5^
Minerals			
Orthoclase	0.8186	− 9.5192 × 10^–3^	1.3965 × 10^–4^
Albite	0.7920	− 9.1116 × 10^–3^	1.3134 × 10^–4^
Quartz	0.7707	− 8.4388 × 10^–3^	1.1769 × 10^–4^
Kaolinite	0.5307	− 3.9962 × 10^–3^	4.6345 × 10^–5^
Forsterite	0.4865	− 3.0535 × 10^–3^	3.4231 × 10^–5^
Montmorillonite	0.4597	− 3.0245 × 10^–3^	3.2987 × 10^–5^
Gibbsite	0.4599	− 3.6652 × 10^–3^	4.1035 × 10^–5^
Cassiterite	0.3884	− 1.5015 × 10^–3^	1.2383 × 10^–5^
Aragonite	0.3606	− 3.0339 × 10^–3^	4.8428 × 10^–5^
Sphalerite	0.3250	− 1.0477 × 10^–3^	5.1003 × 10^–6^
Calcite	0.3278	− 1.9878 × 10^–3^	3.3043 × 10^–5^
Gas species			
Ga_2_O	0.1806	− 1.8980 × 10^–3^	2.8441 × 10^–5^
Ga_2_O_3_	0.6763	− 1.4769 × 10^–2^	2.8909 × 10^–4^
Ga_2_S_3_	0.3831	− 3.6826 × 10^–3^	4.0031 × 10^–5^
GaCl_3_	0.4612	− 2.8840 × 10^–3^	2.2142 × 10^–5^
GaF_3_	0.8098	− 1.2551 × 10^–2^	1.8818 × 10^–4^
GaO	0.2220	− 3.4406 × 10^–3^	4.9192 × 10^–5^

#### The 10^3^ln(RPFR)s of Ga-bearing minerals

The eleven Ga-bearing minerals (orthoclase, albite, quartz, kaolinite, forsterite, montmorillonite, gibbsite, cassiterite, aragonite, sphalerite and calcite) have 10^3^ln(RPFR)s varying from 3.48 to 8.21 ‰ at room temperature (25 °C). The specific data of 10^3^ln(RPFR)s for orthoclase, albite, quartz, kaolinite, forsterite, montmorillonite, gibbsite, cassiterite, aragonite, sphalerite and calcite in the temperature range of 0–300 °C can be obtained from Table 1. The 10^3^lnRPFR values of two different caution cites of forsterite at different temperatures were shown in Table S3. The scaling factors were not used for all eleven minerals.

#### Fractionation factors of Ga-bearing minerals

**Figure 5:**
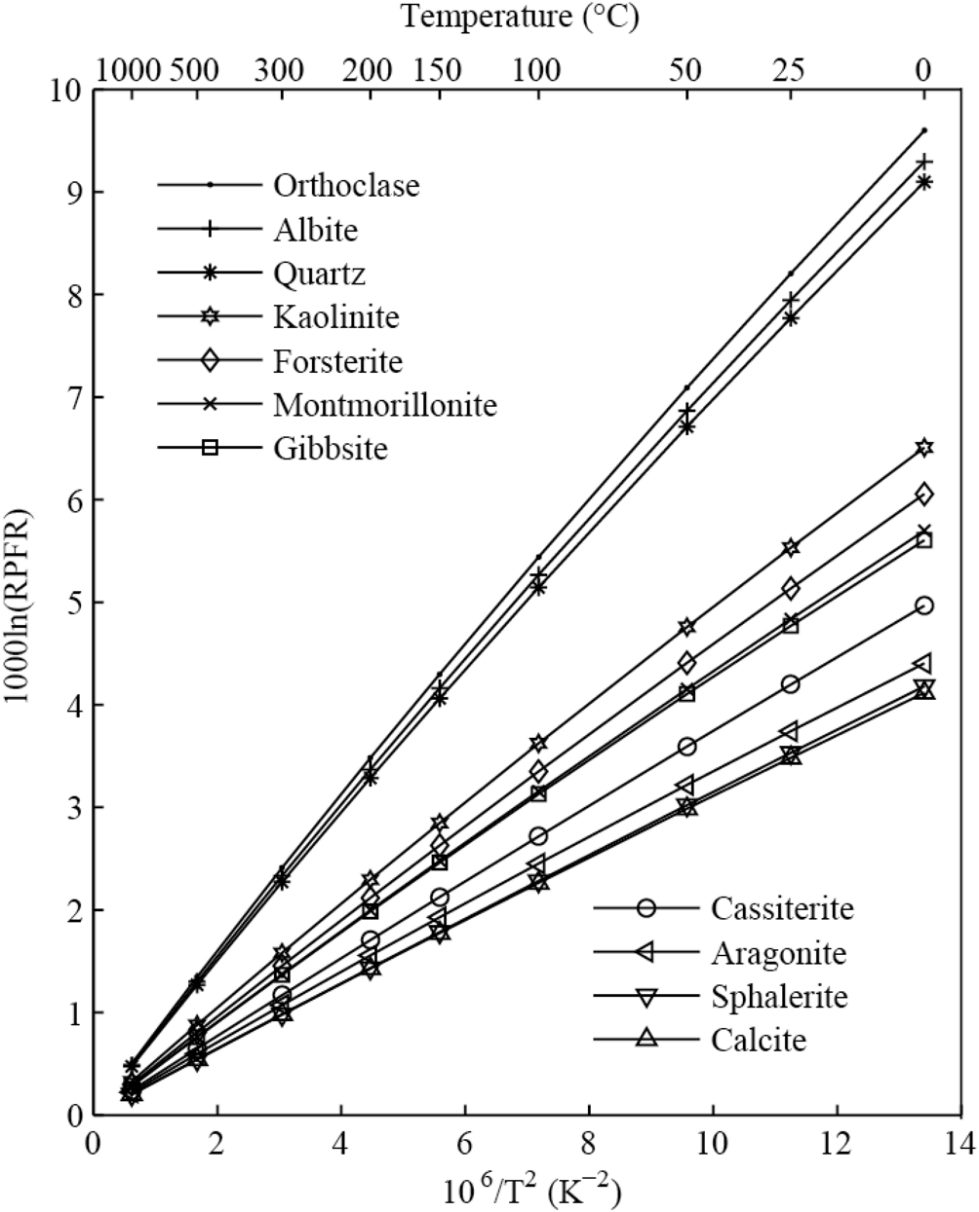
The graphical representation of 10^3^ln(RPFR)s for different Ga-bearing minerals vs temperature. The basis set of B3LYP/6-311 + G(d) is used for all minerals.

**Figure 6:**
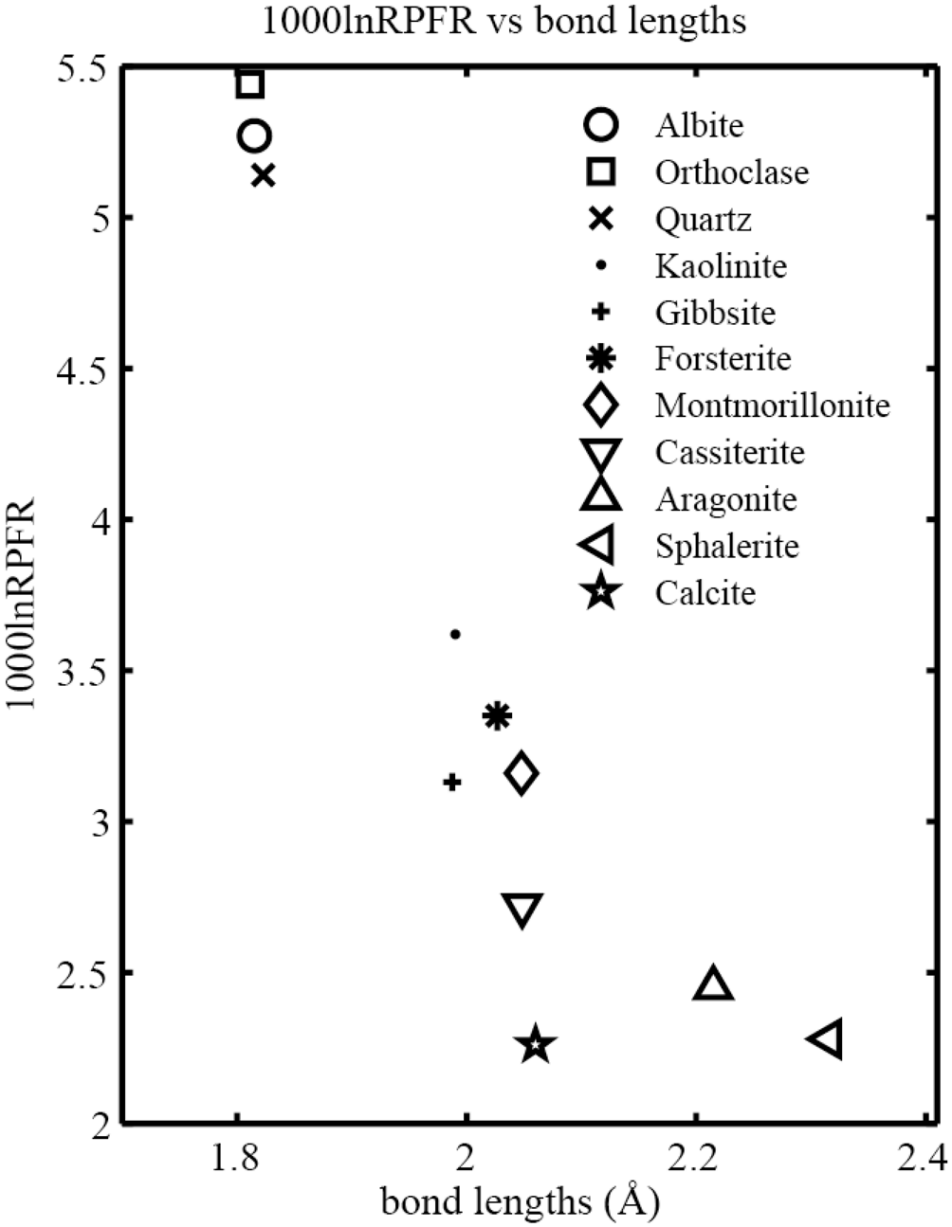
The relationship between 10^3^ln(RPFR)s (100 °C) of different Ga-bearing minerals with bond lengths.

Taking the 10^3^ln(RPFR) values of different Ga-bearing minerals at 25 °C as an example, the maximum isotopic fractionation values between these minerals can reach 4.73 ‰ (isotopic fractionation factors between orthoclase and calcite). The central atoms of these different solid minerals were substituted by Ga as research objects. The results show that the Ga isotope enrichment sequence for these minerals is: orthoclase > albite > quartz > kaolinite > forsterite > montmorillonite > gibbsite > cassiterite > aragonite > sphalerite > calcite. Ga isotope fractionation between these minerals decreases with increasing temperature. The isotopic fractionation factor between orthoclase and calcite can reach 3.18 per mil even at 100 °C. Therefore, the isotopic fractionation of Ga isotopes can be significantly fractionated between these Ga-bearing minerals. The 10^3^ln(RPFR)s of these 11 Ga-bearing minerals varies with temperature, as shown in Fig. 5. Among these Ga-bearing minerals, silicate minerals show the strongest ability to enrich heavy Ga isotopes (^71^ Ga). This ability is related to the strength of the chemical bonds formed between Ga and negatively charged atoms (Fig. 6). In other words, the chemical bonds formed between aluminosilicate and Ga are strongest.

#### The 10^3^ln(RPFR)s of Ga-bearing gas phases

**Figure 7:**
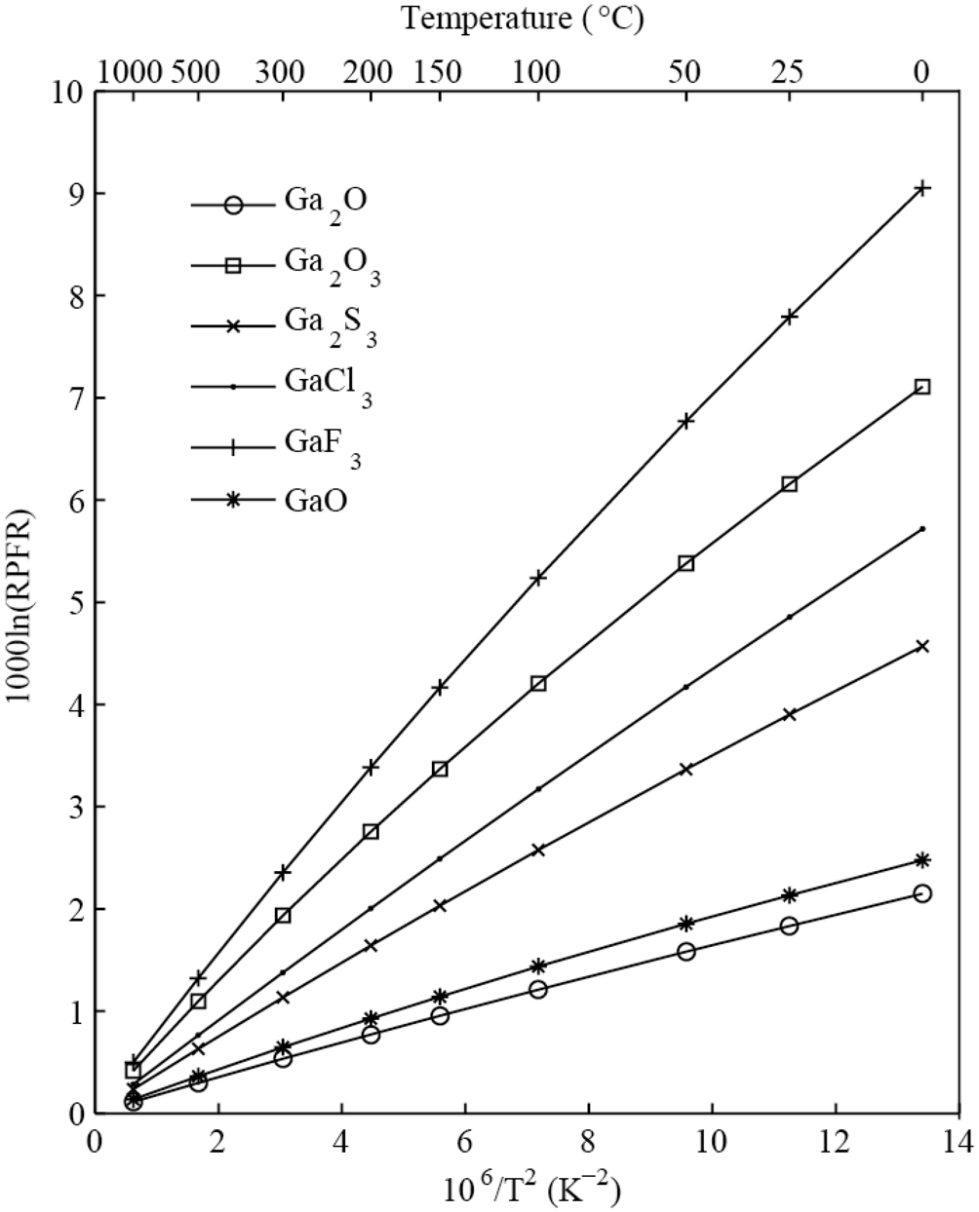
The l0^3^ln(RPFR)s of Ga_2_O, Ga_2_O_3_, Ga_2_S_3_, GaCl_3_, GaF_3_ and GaO as a function of temperature at the basis set of B3LYP/6-311 + G(d) level.

Ga_2_O, Ga_2_O_3_, Ga_2_S_3_, GaCl_3_, GaF_3_ and GaO, were chosen to represent the isotope compositions of Ga-bearing gas phases. Table 1 shows the 10^3^ln(RPFR) values of potential Ga-bearing gas phases at different temperatures. Table 2 presents the polynomial expansions of the RPFR factors (^71^Ga/^69^Ga) of different Ga-bearing species based on the function 10^3^lnRPFR = ax + bx^2^ + cx^3^, with x = 10^6^/T^2^ (T is the temperature in Kelvin). Figure 7 illustrates the 10^3^ln(RPFR) values of different Ga-bearing gas species as a function of temperature. The scaling factors were not used for the six gas phases.

#### The 10^3^ln(α)s for pairs of Kln-solutions

In this study, the isotopic fractionation factors between kaolinite (Kln) and aqueous solutions are given in Table 3. The Ga isotope fractionation factors between other Ga-bearing minerals and aqueous solutions can also be obtained by using this method and shown in Table S4. The general trend of isotopic fractionation between kaolinite and aqueous solution is that the Ga-bearing aqueous solutions are enriched in heavy isotopes relative to kaolinite. The Ga fractionation factors for 5 Kln-solution pairs is decreasing in the order of Kln-[Ga(H_2_O)_6_]^3+^·(H_2_O)_24_ > Kln-[Ga(OH)_2_]^+^ (H_2_O)_24_ > Kln-[Ga(OH)]^2+^·(H_2_O)_24_ > Kln-[Ga(OH)_3_]·(H_2_O)_24_ > Kln-[Ga(OH)_4_]^−^·(H_2_O)_24_ at the same temperature.

**Table 3:** The calculated 1000*ln(α) values (^71^ Ga/^69^ Ga) for pairs of Kln-[Ga(H_2_O)_6_]^3+^·(H_2_O)_24_, Kln-[Ga(OH)_2_]^+^·(H_2_O)_24_, Kln-[Ga(OH)]^2+^·(H_2_O)_24_, Kln-[Ga(OH)_3_]·(H_2_O)_24_ and Kln-[Ga(OH)_4_]^−^·(H_2_O)_24_ at different temperatures.

Kln-solution pairs	Temperature (℃)								
	0	25	50	100	150	200	300	500	1000
Kln-[Ga(H_2_O)_6_]^3+^·(H_2_O)_24_	− 0.4	− 0.36	− 0.31	− 0.25	− 0.2	− 0.16	− 0.12	− 0.07	− 0.02
Kln-[Ga(OH)_2_]^+^·(H_2_O)_24_	− 0.47	− 0.43	− 0.38	− 0.31	− 0.25	− 0.21	− 0.16	− 0.09	− 0.03
Kln-[Ga(OH)]^2+^·(H_2_O)_24_	− 0.72	− 0.64	− 0.56	− 0.45	− 0.36	− 0.3	− 0.21	− 0.12	− 0.04
Kln-[Ga(OH)_3_]·(H_2_O)_24_	− 1.93	− 1.69	− 1.49	− 1.18	− 0.94	− 0.77	− 0.55	− 0.31	− 0.12
Kln-[Ga(OH)_4_]^−^·(H_2_O)_24_	− 2.03	− 1.77	− 1.55	− 1.22	− 0.97	− 0.8	− 0.56	− 0.32	− 0.12

## Discussions

### Ga isotope fractionation in surficial geochemical processes

Ga^3+^ exists in different forms in water with different pH values. As the pH increases, the amount of Ga(OH) increases^28^. In this study, [Ga(OH)_4_]^−^, [Ga(OH)_3_], [Ga(OH)_2_]^+^, [Ga(OH)_2_]^+^ and [Ga(H_2_O)_6_]^3+^ were used to simulate the main Ga^3+^-bearing solutions^28^. These species were chosen as the research systems because they have been determined by high-resolution X-ray absorption fine structure (XAFS) and nuclear magnetic resonance (NMR) spectroscopies^28^. Similar to halide and sulfide aqueous solutions usually formed by other metals (such as Zn), halide and sulfide solutions of Ga can hardly exist in natural systems and can only exist in high-concentration acid solutions or anhydrous environments, which only has theoretical research significance^30–32^. Similar to Al, Ga can strongly hydrolyze in aqueous solution, resulting in its inability to form simple aqueous ions. Theoretically existing halide species [GaCl_4_]^−^ and [GaBr_4_]^−^ were also selected to study the isotopic fractionation characteristics of Ga halides. Isotopic fractionation data for Ga halides are provided in Fig. S3. The species coordinated with H_2_O/OH can coexist within a certain pH range. However, under different pH conditions, one or two species are dominant^28^. In a low pH environment, the dominant species exist in the form of Ga^3+^. In aqueous solution, the species does not exist as a single ion but forms an octahedral structure with six water molecules and forms six Ga–O bonds; the specific form is [Ga(H_2_O)_6_]^3+^. When the pH was higher, the dominant species existed as of [Ga(OH)_4_]^−^. Based on previous experimental results, when the solution does not contain Si, the Ga–O bond length is between 1.96 and 1.94 at low pH (approximately 1–4). At high pH (5–10), the Ga–O bond length is between 1.88 and 1.83^28^. This is consistent with the results obtained in this study, in which the average bond length of Ga–O in [Ga(H_2_O)_6_]^3+^ is approximately 1.99, and the average bond length of [Ga(OH)_4_]^−^ is approximately 1.86. The detailed structural information can be seen in Fig. 2, Fig. S2 and Table S2.

To ensure the accuracy of the calculation, four parallel calculations were conducted for each Ga^3+^-bearing aqueous species. The initial structures used for these four parallel calculations are completely different. Thus, a series of structural and frequency information surrounded by 6, 12, 18 and 24 water molecules were obtained (Table S5). The results of this study show that there are slight differences in the structures of the four configurations, resulting in slight differences in their RPFRs. According to the previous research results^33^, when the number of outer water molecules increases to 30, the RPFR of the water molecule cluster tends to converge. That is, the RPFR of 24 water molecules showed the same value as the RPFR of 30 water molecules. Therefore, the structures with 24 water molecules were chosen as the final structure of Ga-bearing solutions for this research.

The RPFR values of these different aqueous solutions are different under the same temperature conditions. Specifically, under alkaline conditions, [Ga(OH)_4_]^−^ is the dominant aqueous species with the largest isotopic fractionation value. Under acidic conditions, [Ga(H_2_O)_6_]^3+^ is the main aqueous species with the smallest isotopic fractionation value. At the same temperature, the change order of RPFR factors for these Ga-bearing aqueous solutions is [Ga(OH)_4_]^−^ > [Ga(OH)_3_] > [Ga(OH)]^2+^ > [Ga(OH)_2_]^+^ > [Ga(H_2_O)_6_]^3+^. Taking Ga isotope fractionation values between different Ga-bearing aqueous solutions at 100 ℃ as an example, the maximum value appears between [Ga(OH)_4_]^−^·(H_2_O)_24_ and [Ga(H_2_O)_6_]^3+^·(H_2_O)_24_, and the fractionation value can reach 0.97 per mil. As temperatures rise, the isotopic fractionation between these species decreases. At 500 ℃, this value drops rapidly to approximately 0.25 per mil. The 10^3^ln(RPFR) values of these Ga-bearing aqueous solutions are listed in Table 1.

Because kaolinite, montmorillonite, gibbsite, cassiterite, aragonite, sphalerite and calcite are potential Ga-bearing minerals in rocks and soils in the crust, isotope enrichments based on these minerals have been used in estimating Ga isotope fractionation in surficial geochemical processes. In this study, Ga substitution for metal ions in these mineral lattice sites forms Ga-bearing mineral structures. A growing number of field and experimental observations indicate that large Ga isotope fractionations can be produced during chemical weathering processes^8,9^. Yuan et al. (2022) demonstrated that Ga isotope fractionation between solution and weathered basalt could reach up to 1.50‰^8^ and concluded that the solution was more isotopically enriched (^71^Ga) than that of the rocks. In addition, Liu et al. (2022) work revealed that soil samples are enriched ^71^Ga relative to bedrock, and the adsorption process can also produce obvious Ga isotope fractionation^9^. For the equilibrium Ga isotope fractionation between these Ga-bearing minerals and the solutions, the solution phases tend to enrich ^71^Ga relative to minerals. This result is also consistent with previous research suggesting that surface river systems are more enriched in heavy Ga isotopes^8^. The relationship between isotope fractionation values and the temperature of potential Ga-bearing minerals and aqueous solutions are presented in Table S4 with a temperature range from 0 to 1000 °C. Although surficial geochemistry is very complex and many factors, such as chemical weathering and biological action, can affect Ga isotope fractionation, the equilibrium Ga isotope fractionation presented in this study can essentially explain the isotope enrichment mechanism among these species.

**Figure 8:**
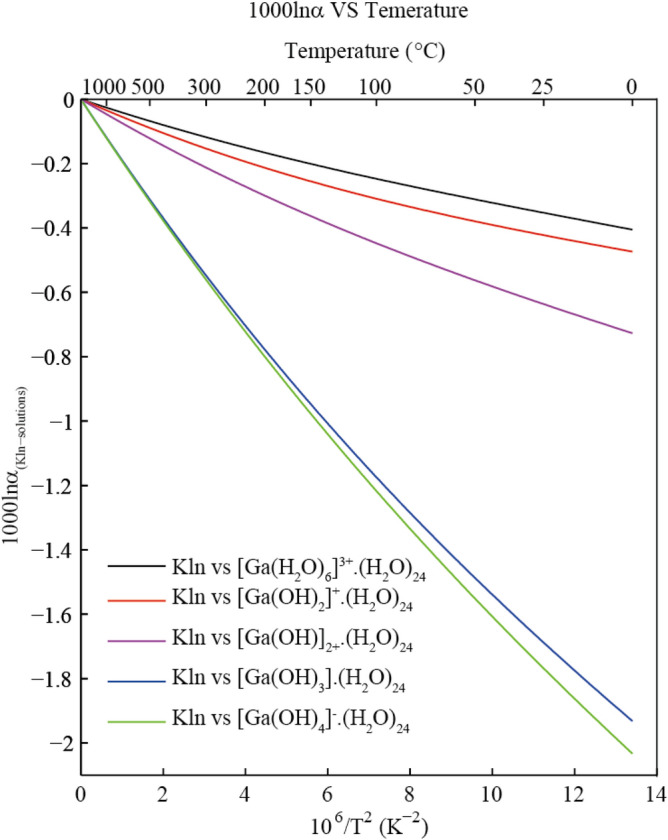
The 10^3^ln(α) (^71^Ga/^69^Ga) of different Kln-solution pairs as function of temperature.

Isotope exchange reactions between minerals and hydrothermal fluids are also very common and are a key factor when determining isotope compositions. The equilibrium isotope fractionation between kaolinite and solutions shows that Ga-bearing aqueous solutions exhibit a stronger ability to enrich heavy isotopes than that of kaolinite. The 10^3^lnα values of Kln-[Ga(H_2_O)_6_]^3+^·(H_2_O)_24_, Kln-[Ga(OH)_2_]^+^·(H_2_O)_24_, Kln-[Ga(OH)]^2+^·(H_2_O)_24_, Kln-[Ga(OH)_3_]·(H_2_O)_24_ and Kln-[Ga(OH)_4_]^−^·(H_2_O)_24_ are no more than − 0.8 per mil at 200 °C (Table 3). The relationship between 10^3^ln(α) (^71^Ga/^69^Ga) and temperature for these Kln-solution pairs is shown in Fig. 8. The theoretical results show that the isotopic fractionations between kaolinite and Ga-bearing solutions are affected by pH. The theoretical prediction results are in agreement with previous experimental observations. Pokrovski et al. (2002) suggested that Ga forms different coordination compounds with H_2_O and/or OH groups in the first coordination sphere of the metal as the pH changes^28^. The main Ga-bearing aqueous solutions corresponding to pH < 3, 3 < pH < 5 and pH > 8 are [Ga(H_2_O)_6_]^3+^, [Ga(OH)]^2+^ and [Ga(OH)_4_]^−^, respectively. Wen et al.’s research on Ga isotope fractionation in the Xiaoshanba bauxite deposit^34^ shows that the Ga isotope fractionation value (δ^71^Ga) of clay with kaolinite as the main mineral is − 0.22‰ to + 0.19‰ (− 0.04 on average). In addition to the contribution of kaolinite, other related Ga-bearing minerals may also impact these data. This study’s results show the isotope fractionation factors of pairs Kln-[Ga(H_2_O)_6_]^3+^·(H_2_O)_24_, Kln-[Ga(OH)_2_]^+^·(H_2_O)_24_ and Kln-[Ga(OH)]^2+^·(H_2_O)_24_ are − 0.12, − 0.16 and − 0.21, respectively, at 300 °C. The data obtained by this research can provide theoretical support for interpreting these experimental observations. Polynomial expansions of 10^3^ln(α) (^71^Ga/^69^Ga) for different Kln-solution pairs are shown in Table 4.

**Table 4:** Polynomial expansions of the 1000ln(α) values (^71^Ga/^69^Ga) for different Ga-bearing species pairs based on the function 10^3^lnβ = ax + bx^2^ + cx^3^, with x = 10^6^/T^2^ (T is the temperature in Kelvin).

Kln-solution pairs	a	b	c
Kln-[Ga(H_2_O)_6_]^3+^·(H_2_O)_24_	− 0.0427	1.4125 × 10^–3^	− 3.5952 × 10^–5^
Kln-[Ga(OH)_2_]^+^·(H_2_O)_24_	− 0.0569	2.3001 × 10^–3^	− 5.1498 × 10^–5^
Kln-[Ga(OH)]^2+^·(H_2_O)_24_	− 0.0764	2.3241 × 10^–3^	− 5.0030 × 10^–5^
Kln-[Ga(OH)_3_]·(H_2_O)_24_	− 0.1941	4.8698 × 10^–3^	− 8.5145 × 10^–5^
Kln-[Ga(OH)_4_]^−^·(H_2_O)_24_	− 0.1968	4.3175 × 10^–3^	− 7.0995 × 10^–5^

### Ga isotope fractionation data during the high-temperature evaporation process

In solar systems, feldspar (including anorthite, albite, and orthoclase) is the main host of meteoritic minerals for Ga^6^. Thus, these minerals are selected to investigate Ga isotope fractionation in the evaporation process.

Six Ga oxides, sulfides and halogen compounds were selected as potential Ga-bearing gaseous species. Among these gaseous species, Ga fluoride has the biggest RPFR value (Fig. 7). There are three kinds of Ga oxides, namely, Ga_2_O, GaO and Ga_2_O_3_. Among these three substances, Ga exhibits different oxidation states, which are + 1, + 2 and + 3. The calculation results show that the higher the oxidation state is, the stronger the ability to enrich ^71^Ga. This is consistent with the general law of isotope enrichment. That is, the higher the valence state is, the stronger the chemical bond strength. In other words, Ga_2_O_3_ shows the strongest ability to enrich ^71^Ga. As an example, for the equilibrium isotope fractionation data (10^3^lnRPFR) of these three gas species at 100 ℃, the values are 1.21, 1.44 and 4.20, respectively. For Ga halides, as the atomic mass of halogen elements increases, the ability to enrich ^71^Ga decreases gradually. In this study, compared to GaCl_3_, GaF_3_ is obviously more likely to enrich heavy isotopes. As mentioned above, Ga is a typical medium volatile element, and its 50% condensation mass temperature is 968 K. Through this theoretical study, it was found that the equilibrium isotope fractionation value of GaF_3_ can still reach approximately 0.5 per mil even when the temperature reaches 1000 °C. Table 1 shows Ga isotope fractionation data at different temperatures (0, 25, 50, 100, 150, 200, 300, 500, and 1000 °C).

**Figure 9:**
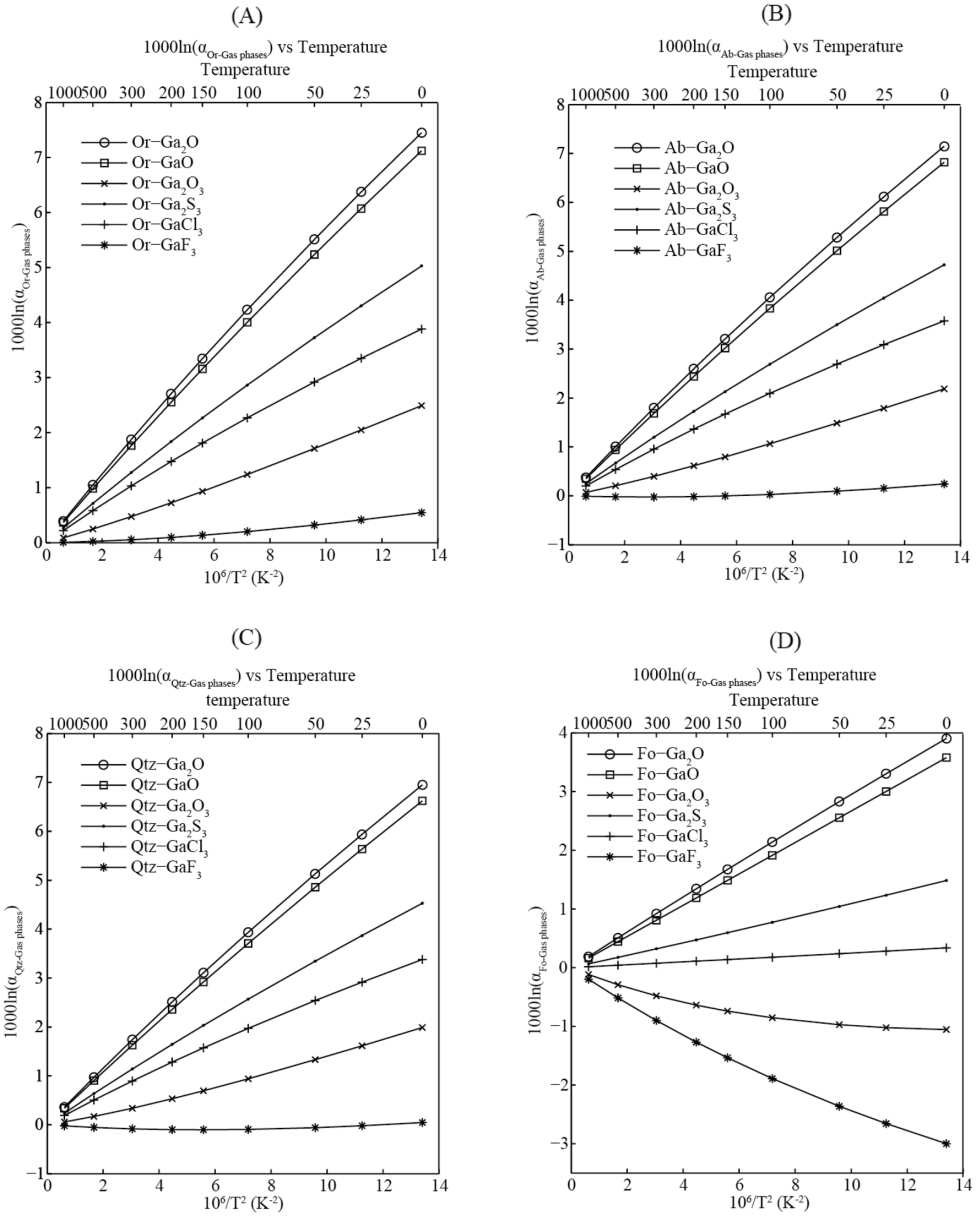
The equilibrium Ga isotope fractionation factors between Ga-bearing minerals (**A**): orthoclase, (**B**): albite, (**C**): quartz and (**D**): forsterite) and gas phases as a function of temperature. Or, Ab, Qtz and Fo are short for orthoclase, albite, quartz and forsterite, respectively.

When studying the isotopic fractionation of the vaporization process, an important step is to accurately determine the morphology of evaporation species. At 1000℃, 10^3^ln(RPFR)s for orthoclase, Ga_2_O and GaO are 0.50‰, 0.11‰ and 0.14‰, respectively. Under these conditions, the maximum of equilibrium Ga isotope fractionation factor (10^3^lnα) is 0.39‰. The equilibrium isotope fractionation factors between minerals and gas phases as a function of temperature are shown in Fig. 9 and Table S6. For pairs of Orthoclase -gas phases and Albite -gas phases, Orthoclase and Albite are more enriched in heavy isotopes relative to gas phases during the evaporation process. The kinetic effect of vaporization is closely related to the mass difference of vaporized species. For potential gas species Ga_2_O, GaO, Ga_2_O_3_, Ga_2_S_3_, GaCl_3_ and GaF_3_, the kinetic isotope fractionation factors (10^3^lnα) are 6.5‰, 11.7‰, 5.4‰, 4.3‰, 5.7‰ and 7.9‰ according to the formula α = (m_H_/m_L_)^0.5^, where m_H_ and m_L_ are the molecular masses with heavy and light isotope atoms, respectively^18^. The influence of the equilibrium process on Ga isotopes is very small compared with that of the kinetic process.

A previous study showed that calcium–aluminum-rich inclusions (CAIs) are enriched in ^71^Ga compared to the bulk meteorite, and the isotope fractionation value could reach up to − 3.56‰ (δ^71^Ga)^12^. The researchers interpreted the large enrichment in the light isotopes of Ga in the CAIs as the introduced of Ga in the CAIs during condensation in the solar nebula rather than secondary processing in the meteorite parent body. Despite the complexity of the process, the results of this study can still provide appropriate theoretical support. According to the predicted RPFRs (Table 1), the equilibrium Ga isotope fractionation factors between Ga-bearing solutions and orthoclase are less than − 2.69‰ (0 °C). At high temperatures, these values become even smaller. In terms of the isotope enrichment mechanism, light Ga isotopes are enriched in solution phases compared with orthoclase. Therefore, Kato and Moynier’s results may hold.

## Conclusions

In this study, the equilibrium isotope fractionation factors between several important Ga isotope systems were estimated with the first-principles DFT method (B3LYP/6-311+G(d)). The results show that the Ga isotope fractionations are significant in a series of different geochemical processes. This study predicts that different Ga-bearing species possess different isotopic compositions. The data obtained in this study can provide theoretical support for investigating different geochemical processes. The large enrichment in the light isotopes of Ga in the CAIs is because the introduced of Ga in the CAIs during condensation in the solar nebula. In the process of rock weathering, Ga-bearing solutions are enriched ^71^Ga relative to minerals such as kaolinite. The investigation of Ga isotopes in this study can provide additional geochemical constraints on these different geochemical processes. The equilibrium Ga isotope fractionation factors predicted in this study could provide some help for future research.

### Calculation methods

Mass-dependent equilibrium isotope fractionation factors are obtained based on the difference in vibration frequencies of different isotopologues^35,36^. Through the Bigeleisen–Mayer equation (Urey model), the equilibrium isotope exchange constant K could be obtained through partition functions of reactants and products. Take a simple isotope exchange reaction as an example:

1 $${}^{69}GaA + {}^{71}GaB = {}^{71}GaA + {}^{69}GaB$$

where Ga is the metal cation gallium, A and B stand for two kinds of anions or ionic groups, and ^71^GaA and ^71^GaB are substances with heavier isotopes relative to ^69^GaA and ^69^GaB, respectively. In quantum mechanics, the reaction equilibrium constant K can be expressed as the ratio of the reduced partition function ratio (RPFR) of these two substances or two different phases of the same substance as follows:

2 $$K_{GaA - GaB} = \frac{{RPFR\left( {GaA} \right)}}{{RPFR\left( {GaB} \right)}}$$

Unlike chemical thermodynamics, the isotope fractionation factor α, in addition to the equilibrium constant K, is widely used in the field of geochemistry. There is a good relationship between α and K, i.e., α = K^1/n^. In the previous formula, “n” is the number of atoms exchanged during the isotopic exchange reaction. If only one atom is exchanged in an isotopic exchange reaction, then α = K. The relationship between the theoretical and experimental values is Δ_GaA-GaB_≈10^3^*lnα (GaA and GaB indicate two different Ga-bearing substances). The calculation formula of RPFR is as follows:

3 $$\left( {\frac{s}{s*}} \right)RPFR = \prod\limits_{i}^{3n - 6} {\frac{{u_{i} }}{{u_{i} *}}\frac{{\exp \left( {{{ - u_{i} } \mathord{\left/ {\vphantom {{ - u_{i} } 2}} \right. \kern-0pt} 2}} \right)}}{{\exp \left( {{{ - u_{i}^{*} } \mathord{\left/ {\vphantom {{ - u_{i}^{*} } 2}} \right. \kern-0pt} 2}} \right)}}\frac{{1 - \exp \left( { - u_{i} *} \right)}}{{1 - \exp \left( { - u_{i} } \right)}}}$$

where “s” is the symmetry number of the research system. For most complex systems, “s” is equal to “s*”. The symbols marked with asterisks correspond to the molecules with heavier isotopes. u_i_ is a function of the vibration frequency, which can be obtained through the following equation:

4 $$u_{i} { = }\frac{{{\text{h}}\nu_{{\text{i}}} }}{{{\text{k}}_{{\text{b}}} {\text{T}}}}$$

where h, k_b_ and T stand for the Planck constant, Boltzmann constant and temperature in Kelvin, respectively. Based on the formulas mentioned above, the equilibrium isotope fractionation factor α could be obtained. Once the simple harmonic vibration frequencies of the material are obtained by theoretical calculation, the equilibrium isotope fractionation factor α is just a function of temperature. The magnitude of the equilibrium isotope fractionation factor is inversely proportional to the temperature. This is why the value of isotopic fractionation at low temperatures is larger than that at high temperatures.

Importantly, in the Urey model or Bigeleisen–Mayer equation, simple harmonic vibrational frequencies are used rather than the experimental fundamental frequencies^37^. Using ab initio or first-principles methods to calculate isotope fractionation factors has become a routine job^19–26,33,38–52^. A scaling treatment is usually necessary to obtain a more reasonable harmonic vibration frequency^53^. However, in this study, scaling factors are not used from beginning to end. This is because when the scaling factor is used to correct the frequencies of light and heavy isotopologues, this correction is also negated when RPFR is calculated using the Bigeleisen–Mayer equation. Since the Bigeleisen–Mayer equation appeared, theoretical research on this equation involves an endless stream^37,54,55^. In this study, Gaussian16 software is used for all calculations^56^.

The VVCM method was used to simulate solid minerals. This approach has been reported in numerous studies^33,38,52,57,58^, demonstrating that the theoretical calculation method adopted by this study is feasible and reasonable. A previous study indicated that the chemical environment of minerals could be simulated by molecular clusters^59^. When constructing the mineral molecular clusters, the atoms of interest must be placed at the center of the mineral fragment. In this study, all atoms in the center are Ga. The basis set of B3LYP/6-311+G(d) is used for all Ga-bearing minerals in this study. When dealing with the solvation effect, the water-droplet method was used. The details of the calculation method are shown in supplementary file.

## Supplementary Information

Supplementary Information.

## Data Availability

The datasets generated and/or analysed during the current study are not publicly available due to privacy restrictions but are available from the corresponding author on reasonable request.
