# Supplementary material for: First-principles calculations of equilibrium Ga isotope fractionations between several important Ga-bearing minerals and aqueous solutions
Source: Sci Rep. 2023 Apr 17;13:6230. doi: 10.1038/s41598-023-32858-0 (PMC10110521; doi:10.1038/s41598-023-32858-0)
Supplement: Supplementary file 1 — Supplementary Information. [file 41598_2023_32858_MOESM1_ESM.docx]

**Tables**

**Table S1.** The bond lengths and coordination numbers of the Ga-bearing minerals. The numbers and the letter N represent the serial number of the bonds and anions such as O and S, respectively.

| Bond lengths  Minerals | Ga-N | | | | | | |
| --- | --- | --- | --- | --- | --- | --- | --- |
|  | 1 | 2 | 3 | 4 | 5 | 6 | Average |
| Albite | 1.81 | 1.83 | 1.80 | 1.80 |  |  | 1.81 |
| Calcite | 2.07 | 2.07 | 2.07 | 2.05 | 2.05 | 2.07 | 2.06 |
| Aragonite | 2.15 | 3.06 | 2.00 | 2.19 | 1.88 | 1.98 | 2.21 |
| Cassiterite | 2.06 | 2.05 | 2.06 | 2.07 | 2.02 | 2.02 | 2.05 |
| Forsterite | 2.04 | 2.04 | 2.03 | 2.03 | 2.01 | 2.01 | 2.03 |
| Gibbsite | 1.95 | 2.02 | 2.00 | 1.99 | 1.98 | 1.98 | 1.99 |
| Kaolinite | 2.05 | 2.00 | 1.96 | 1.95 | 1.98 | 1.98 | 1.99 |
| Montmorillonite | 2.11 | 2.09 | 2.14 | 2.11 | 1.91 | 1.92 | 2.05 |
| Orthoclase | 1.83 | 1.79 | 1.80 | 1.82 |  |  | 1.81 |
| Quartz | 1.84 | 1.82 | 1.82 | 1.82 |  |  | 1.82 |
| Sphalerite | 2.31 | 2.31 | 2.31 | 2.31 |  |  | 2.31 |

**Table S2.** The structural parameters (bond lengths and coordination numbers) of the Ga-bearing solutions.

|  |
| --- |
|  |
|  |
|  |
|  |
|  |

| parameters  solutions | coordination numbers | Bond lengths | | | |
| --- | --- | --- | --- | --- | --- |
|  |  | Ga-O (H2O) | | Ga-O (OH) | |
|  |  | This study | Exp^a^ | This study | Exp^a^ |
| [Ga(H2O)_6_]^3+^.(H_2_O)_24_ | 6 | 1.99 | 1.94-1.96 |  |  |
| [Ga(OH)_4_]^-^.(H_2_O)_24_ | 4 |  |  | 1.86 | 1.83-1.88 |
| [Ga(OH)_3_].(H_2_O)_24_ | 4 | 1.94 |  | 1.85 |  |
| [Ga(OH)_2_]^+^.(H_2_O)_24_ | 6 | 2.06 |  | 1.89 |  |
| [Ga(OH)]^2+^.(H_2_O)_24_ | 6 | 1.99 |  | 1.86 |  |

Note: “^a^” stands for the reference of POKROVSKI et al. (2002).

**Table S3.** The 10^3^lnRPFR values of two different caution cites (M1 and M2) of forsterite at different temperatures.

| Temperatures(℃)  structures | 0 | 25 | 50 | 100 | 150 | 200 | 300 | 500 | 1000 |
| --- | --- | --- | --- | --- | --- | --- | --- | --- | --- |
| Forsterite-M1 | 6.05 | 5.13 | 4.40 | 3.34 | 2.62 | 2.11 | 1.45 | 0.80 | 0.29 |
| Forsterite-M2 | 5.16 | 4.36 | 3.74 | 2.83 | 2.22 | 1.78 | 1.22 | 0.67 | 0.25 |

**Table S4.** The relationship between isotope fractionation values and the temperature of potential Ga-bearing minerals and aqueous solutions with a temperature range from 0 to 1000℃.

| Temperature  (℃)  mineral-solution pairs | 0 | 25 | 50 | 100 | 150 | 200 | 300 | 500 | 1000 |
| --- | --- | --- | --- | --- | --- | --- | --- | --- | --- |
| Kln-[Ga(H_2_O)_6_]^3+^.(H_2_O)_24_ | -0.4 | -0.36 | -0.31 | -0.25 | -0.2 | -0.16 | -0.12 | -0.07 | -0.02 |
| Kln-[Ga(OH)_2_]^+^.(H_2_O)_24_ | -0.47 | -0.43 | -0.38 | -0.31 | -0.25 | -0.21 | -0.16 | -0.09 | -0.03 |
| Kln-[Ga(OH)]^2+^.(H_2_O)_24_ | -0.72 | -0.64 | -0.56 | -0.45 | -0.36 | -0.3 | -0.21 | -0.12 | -0.04 |
| Kln-[Ga(OH)_3_].(H_2_O)_24_ | -1.93 | -1.69 | -1.49 | -1.18 | -0.94 | -0.77 | -0.55 | -0.31 | -0.12 |
| Kln-[Ga(OH)_4_]^-^.(H_2_O)_24_ | -2.03 | -1.77 | -1.55 | -1.22 | -0.97 | -0.8 | -0.56 | -0.32 | -0.12 |
| Mon-[Ga(H_2_O)_6_]^3+^.(H_2_O)_24_ | -1.21 | -1.05 | -0.91 | -0.71 | -0.57 | -0.46 | -0.32 | -0.18 | -0.07 |
| Mon-[Ga(OH)_2_]^+^.(H_2_O)_24_ | -1.28 | -1.12 | -0.98 | -0.77 | -0.62 | -0.51 | -0.36 | -0.21 | -0.08 |
| Mon-[Ga(OH)]^2+^.(H_2_O)_24_ | -1.54 | -1.33 | -1.17 | -0.91 | -0.73 | -0.60 | -0.42 | -0.24 | -0.09 |
| Mon-[Ga(OH)_3_].(H_2_O)_24_ | -2.74 | -2.38 | -2.09 | -1.64 | -1.31 | -1.07 | -0.75 | -0.43 | -0.16 |
| Mon-[Ga(OH)_4_]^-^.(H_2_O)_24_ | -2.84 | -2.46 | -2.15 | -1.68 | -1.34 | -1.10 | -0.77 | -0.43 | -0.16 |
| Gib-[Ga(H_2_O)_6_]^3+^.(H_2_O)_24_ | -1.31 | -1.12 | -0.96 | -0.74 | -0.58 | -0.47 | -0.33 | -0.18 | -0.07 |
| Gib-[Ga(OH)_2_]^+^.(H_2_O)_24_ | -1.37 | -1.18 | -1.03 | -0.80 | -0.64 | -0.52 | -0.36 | -0.21 | -0.08 |
| Gib-[Ga(OH)]^2+^.(H_2_O)_24_ | -1.63 | -1.40 | -1.21 | -0.94 | -0.75 | -0.61 | -0.42 | -0.24 | -0.09 |
| Gib-[Ga(OH)_3_].(H_2_O)_24_ | -2.83 | -2.45 | -2.14 | -1.66 | -1.33 | -1.09 | -0.76 | -0.43 | -0.16 |
| Gib-[Ga(OH)_4_]^-^.(H_2_O)_24_ | -2.93 | -2.53 | -2.20 | -1.71 | -1.36 | -1.11 | -0.77 | -0.43 | -0.16 |
| Cas-[Ga(H_2_O)_6_]^3+^.(H_2_O)_24_ | -1.94 | -1.69 | -1.47 | -1.15 | -0.92 | -0.75 | -0.53 | -0.30 | -0.11 |
| Cas-[Ga(OH)_2_]^+^.(H_2_O)_24_ | -2.016 | -1.76 | -1.54 | -1.21 | -0.98 | -0.80 | -0.56 | -0.32 | -0.12 |
| Cas-[Ga(OH)]^2+^.(H_2_O)_24_ | -2.27 | -1.97 | -1.73 | -1.35 | -1.08 | -0.89 | -0.62 | -0.35 | -0.13 |
| Cas-[Ga(OH)_3_].(H_2_O)_24_ | -3.47 | -3.02 | -2.65 | -2.08 | -1.67 | -1.36 | -0.96 | -0.54 | -0.20 |
| Cas-[Ga(OH)_4_]^-^.(H_2_O)_24_ | -3.57 | -3.10 | -2.71 | -2.12 | -1.70 | -1.38 | -0.97 | -0.55 | -0.21 |
| Ara-[Ga(H_2_O)_6_]^3+^.(H_2_O)_24_ | -2.51 | -2.14 | -1.85 | -1.42 | -1.12 | -0.91 | -0.68 | -0.35 | -0.13 |
| Ara-[Ga(OH)_2_]^+^.(H_2_O)_24_ | -2.57 | -2.21 | -1.92 | -1.48 | -1.17 | -0.95 | -0.66 | -0.37 | -0.14 |
| Ara-[Ga(OH)]^2+^.(H_2_O)_24_ | -2.83 | -2.42 | -2.10 | -1.62 | -1.28 | -1.04 | -0.72 | -0.40 | -0.15 |
| Ara-[Ga(OH)_3_].(H_2_O)_24_ | -4.03 | -3.48 | -3.02 | -2.34 | -1.86 | -1.51 | -1.05 | -0.59 | -0.22 |
| Ara-[Ga(OH)_4_]^-^.(H_2_O)_24_ | -4.13 | -3.56 | -3.09 | -2.39 | -1.89 | -1.54 | -1.07 | -0.60 | -0.22 |
| Sph-[Ga(H_2_O)_6_]^3+^.(H_2_O)_24_ | -2.73 | -2.35 | -2.04 | -1.58 | -1.26 | -1.03 | -0.72 | -0.40 | -0.15 |
| Sph-[Ga(OH)_2_]^+^.(H_2_O)_24_ | -2.80 | -2.42 | -2.12 | -1.65 | -1.32 | -1.08 | -0.75 | -0.42 | -0.16 |
| Sph-[Ga(OH)]^2+^.(H_2_O)_24_ | -3.05 | -2.64 | -2.30 | -1.79 | -1.42 | -1.16 | -0.81 | -0.46 | -0.17 |
| Sph-[Ga(OH)_3_].(H_2_O)_24_ | -4.26 | -3.69 | -3.22 | -2.51 | -2.01 | -1.64 | -1.15 | -0.65 | -0.24 |
| Sph-[Ga(OH)_4_]^-^.(H_2_O)_24_ | -4.36 | -3.77 | -3.29 | -2.55 | -2.04 | -1.66 | -1.16 | -0.65 | -0.24 |
| Cal-[Ga(H_2_O)_6_]^3+^.(H_2_O)_24_ | -2.79 | -2.40 | -2.08 | -1.60 | -1.27 | -1.03 | -0.72 | -0.40 | -0.15 |
| Cal-[Ga(OH)_2_]^+^.(H_2_O)_24_ | -2.86 | -2.47 | -2.15 | -1.66 | -1.32 | -1.08 | -0.75 | -0.42 | -0.16 |
| Cal-[Ga(OH)]^2+^.(H_2_O)_24_ | -3.12 | -2.68 | -2.33 | -1.80 | -1.43 | -1.16 | -0.81 | -0.45 | -0.17 |
| Cal-[Ga(OH)_3_].(H_2_O)_24_ | -4.32 | -3.73 | -3.25 | -2.53 | -2.02 | -1.64 | -1.14 | -0.64 | -0.24 |
| Cal-[Ga(OH)_4_]^-^.(H_2_O)_24_ | -4.42 | -3.81 | -3.32 | -2.57 | -2.05 | -1.66 | -1.16 | -0.65 | -0.24 |

**Table S5.** 1000lnRPFR values of different Ga-bearing solutions with different number of water molecules at different temperatures.

| Temperature  (℃)  Solutions | 0 | 25 | 50 | 100 | 150 | 200 | 300 | 500 | 1000 |
| --- | --- | --- | --- | --- | --- | --- | --- | --- | --- |

| [Ga(OH)_4_]^-^.(H_2_O)_6_ | 8.49 | 7.26 | 6.28 | 4.82 | 3.81 | 3.08 | 2.13 | 1.19 | 0.62 | 0.44 |
| --- | --- | --- | --- | --- | --- | --- | --- | --- | --- | --- |
| [Ga(OH)_4_]^-^.(H_2_O)_12_ | 8.39 | 7.17 | 6.20 | 4.75 | 3.76 | 3.04 | 2.10 | 1.17 | 0.62 | 0.44 |
| [Ga(OH)_4_]^-^.(H_2_O)_18_ | 8.39 | 7.17 | 6.20 | 4.75 | 3.75 | 3.04 | 2.10 | 1.17 | 0.62 | 0.44 |
| [Ga(OH)_4_]^-^.(H_2_O)_24_ | 8.54 | 7.30 | 6.30 | 4.84 | 3.82 | 3.09 | 2.14 | 1.19 | 0.62 | 0.44 |
| [Ga(H2O)_6_]^3+^.(H_2_O)_6_ | 6.79 | 5.78 | 4.98 | 3.80 | 2.99 | 2.41 | 1.66 | 0.93 | 0.48 | 0.34 |
| [Ga(H2O)_6_]^3+^.(H_2_O)_12_ | 6.84 | 5.82 | 5.01 | 3.82 | 3.01 | 2.43 | 1.68 | 0.93 | 0.49 | 0.34 |
| [Ga(H2O)_6_]^3+^.(H_2_O)_18_ | 6.87 | 5.85 | 5.03 | 3.84 | 3.02 | 2.44 | 1.68 | 0.94 | 0.49 | 0.35 |
| [Ga(H2O)_6_]^3+^.(H_2_O)_24_ | 6.91 | 5.88 | 5.06 | 3.87 | 3.04 | 2.46 | 1.69 | 0.94 | 0.49 | 0.35 |
| [Ga(OH)]^2+^.(H_2_O)_6_ | 7.43 | 6.36 | 5.50 | 4.22 | 3.34 | 2.71 | 1.87 | 1.05 | 0.55 | 0.39 |
| [Ga(OH)]^2+^.(H_2_O)_12_ | 7.30 | 6.23 | 5.37 | 4.11 | 3.25 | 2.62 | 1.81 | 1.01 | 0.53 | 0.37 |
| [Ga(OH)]^2+^.(H_2_O)_18_ | 7.24 | 6.17 | 5.32 | 4.07 | 3.21 | 2.60 | 1.79 | 1.00 | 0.52 | 0.37 |
| [Ga(OH)]^2+^.(H_2_O)_24_ | 7.23 | 6.16 | 5.32 | 4.07 | 3.21 | 2.59 | 1.79 | 1.00 | 0.52 | 0.37 |
| [Ga(OH)_3_].(H_2_O)_6_ | 8.39 | 7.19 | 6.22 | 4.78 | 3.78 | 3.07 | 2.12 | 1.19 | 0.62 | 0.44 |
| [Ga(OH)_3_].(H_2_O)_12_ | 8.33 | 7.13 | 6.16 | 4.73 | 3.74 | 3.03 | 2.10 | 1.17 | 0.61 | 0.44 |
| [Ga(OH)_3_].(H_2_O)_18_ | 8.33 | 7.13 | 6.16 | 4.73 | 3.74 | 3.03 | 2.10 | 1.17 | 0.61 | 0.44 |
| [Ga(OH)_3_].(H_2_O)_24_ | 8.43 | 7.22 | 6.24 | 4.79 | 3.79 | 3.07 | 2.13 | 1.19 | 0.62 | 0.44 |
| [Ga(OH)_2_]^+^.(H_2_O)_6_ | 7.42 | 6.35 | 5.49 | 4.22 | 3.34 | 2.70 | 1.87 | 1.04 | 0.55 | 0.39 |
| [Ga(OH)_2_]^+^.(H_2_O)_12_ | 7.09 | 6.05 | 5.22 | 3.99 | 3.15 | 2.55 | 1.76 | 0.98 | 0.51 | 0.36 |
| [Ga(OH)_2_]^+^.(H_2_O)_18_ | 6.98 | 5.95 | 5.13 | 3.92 | 3.09 | 2.50 | 1.72 | 0.96 | 0.50 | 0.36 |
| [Ga(OH)_2_]^+^.(H_2_O)_24_ | 7.02 | 5.99 | 5.16 | 3.95 | 3.11 | 2.52 | 1.74 | 0.97 | 0.51 | 0.36 |
| [Ga(OH)_2_]^+^.(H_2_O)_30_ | 7.01 | 5.98 | 5.16 | 3.94 | 3.11 | 2.51 | 1.74 | 0.97 | 0.51 | 0.36 |

**Talbe S6.** The equilibrium isotope fractionation factors between minerals and gas phases as a function of temperature, Or, Ab, Qtz and Fo are short for orthoclase, albite, quartz and forsterite, respectively.

| Temperatures(℃)  Mineral-gas pairs | 0 | 25 | 50 | 100 | 150 | 200 | 300 | 500 | 1000 |
| --- | --- | --- | --- | --- | --- | --- | --- | --- | --- |
| Or-Ga_2_O | 7.44 | 6.37 | 5.50 | 4.23 | 3.34 | 2.70 | 1.87 | 1.04 | 0.39 |
| Or-Ga_2_O_3_ | 2.48 | 2.04 | 1.71 | 1.23 | 0.93 | 0.72 | 0.47 | 0.24 | 0.08 |
| Or-Ga_2_S_3_ | 5.02 | 4.30 | 3.72 | 2.86 | 2.26 | 1.83 | 1.27 | 0.71 | 0.26 |
| Or-GaCl_3_ | 3.88 | 3.35 | 2.91 | 2.26 | 1.80 | 1.47 | 1.03 | 0.58 | 0.21 |
| Or-GaF_3_ | 0.54 | 0.41 | 0.31 | 0.20 | 0.13 | 0.09 | 0.05 | 0.02 | 0.01 |
| Or-GaO | 7.12 | 6.07 | 5.23 | 4.00 | 3.15 | 2.55 | 1.76 | 0.98 | 0.36 |
| Ab-Ga_2_O | 7.14 | 6.11 | 5.28 | 4.05 | 3.20 | 2.59 | 1.79 | 1.00 | 0.37 |
| Ab-Ga_2_O_3_ | 2.18 | 1.78 | 1.48 | 1.06 | 0.79 | 0.61 | 0.39 | 0.20 | 0.07 |
| Ab-Ga_2_S_3_ | 4.72 | 4.04 | 3.49 | 2.69 | 2.13 | 1.72 | 1.19 | 0.67 | 0.25 |
| Ab-GaCl_3_ | 3.57 | 3.09 | 2.69 | 2.09 | 1.67 | 1.36 | 0.95 | 0.53 | 0.20 |
| Ab-GaF_3_ | 0.24 | 0.15 | 0.09 | 0.03 | -0.00 | -0.01 | -0.02 | -0.02 | -0.01 |
| Alb-GaO | 6.81 | 5.81 | 5.01 | 3.83 | 3.02 | 2.44 | 1.68 | 0.93 | 0.35 |
| Qtz-Ga_2_O | 6.94 | 5.93 | 5.13 | 3.93 | 3.10 | 2.51 | 1.73 | 0.97 | 0.36 |
| Qtz-Ga_2_O_3_ | 1.98 | 1.61 | 1.33 | 0.94 | 0.69 | 0.53 | 0.33 | 0.17 | 0.06 |
| Qtz-Ga_2_S_3_ | 4.37 | 3.72 | 3.21 | 2.45 | 1.93 | 1.55 | 1.07 | 0.59 | 0.22 |
| Qtz-GaCl_3_ | 3.38 | 2.91 | 2.53 | 1.97 | 1.57 | 1.28 | 0.89 | 0.50 | 0.18 |
| Qtz-GaF_3_ | 0.04 | -0.02 | -0.05 | -0.09 | -0.10 | -0.09 | -0.08 | -0.05 | -0.02 |
| Qtz-GaO | 6.62 | 5.63 | 4.85 | 3.70 | 2.92 | 2.35 | 1.62 | 0.90 | 0.33 |
| Fo-Ga_2_O | 3.90 | 3.30 | 2.82 | 2.13 | 1.67 | 1.34 | 0.92 | 0.50 | 0.18 |
| Fo-Ga_2_O_3_ | -1.05 | -1.02 | -0.97 | -0.85 | -0.73 | -0.63 | -0.48 | -0.29 | -0.11 |
| Fo-Ga_2_S_3_ | 1.48 | 1.23 | 1.04 | 0.77 | 0.59 | 0.47 | 0.32 | 0.17 | 0.06 |
| Fo-GaCl_3_ | 0.33 | 0.28 | 0.23 | 0.17 | 0.13 | 0.11 | 0.07 | 0.04 | 0.01 |
| Fo-GaF_3_ | -2.99 | -2.65 | -2.36 | -1.88 | -1.53 | -1.26 | -0.90 | -0.51 | -0.19 |
| Fo-GaO | 3.57 | 3.00 | 2.55 | 1.91 | 1.48 | 1.18 | 0.80 | 0.44 | 0.16 |

**Figures:**

**Figure S1.** There are two different positions for Mg in forsterite, and these two structures with Mg is replaced by Ga are discussed. M1 and M2 represent these two structures of forsterite, are made up of 99 and 106 atoms respectively.


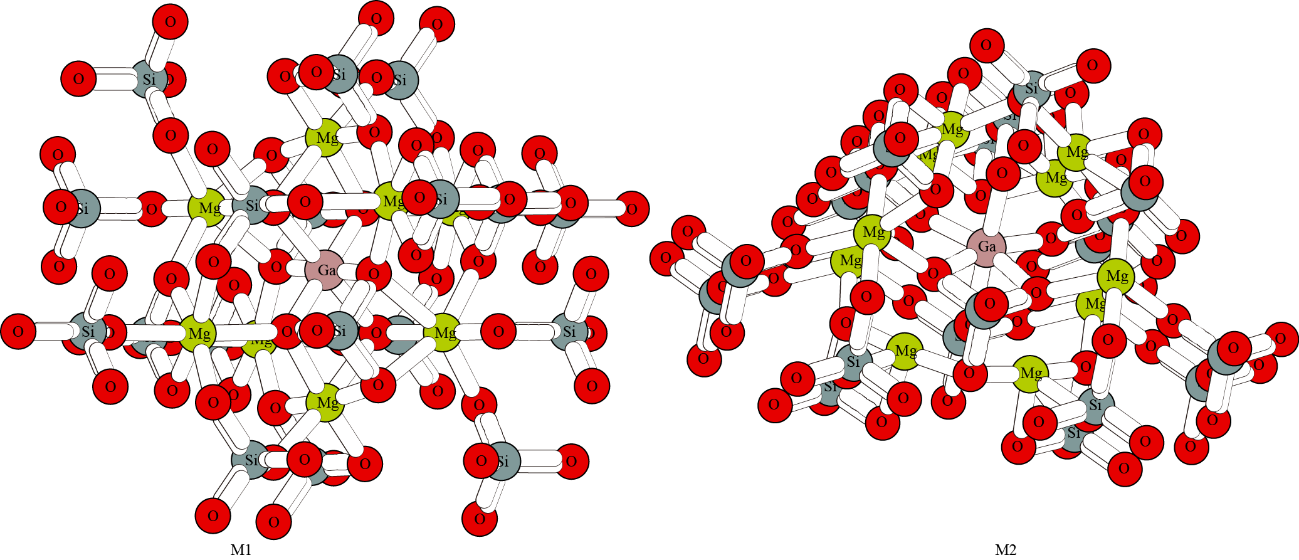


**Figure S2.** The first-coordination shell of different Ga-bearing aqueous solutions. The spatial structure for [Ga(H_2_O)_6_]^3+^, [Ga(OH)]^2+^ and [Ga(OH)_2_]^+^, [Ga(OH)_3_] and [Ga(OH)_4_]^-^ are octahedron, and tetrahedron, respectively. For [Ga(OH)]^2+^, [Ga(OH)_2_]^+^ and [Ga(OH)_3_], the water molecules in the innermost-coordination shell are omitted. The numbers in this figure stand for bond lengths of different Ga-O bonds.


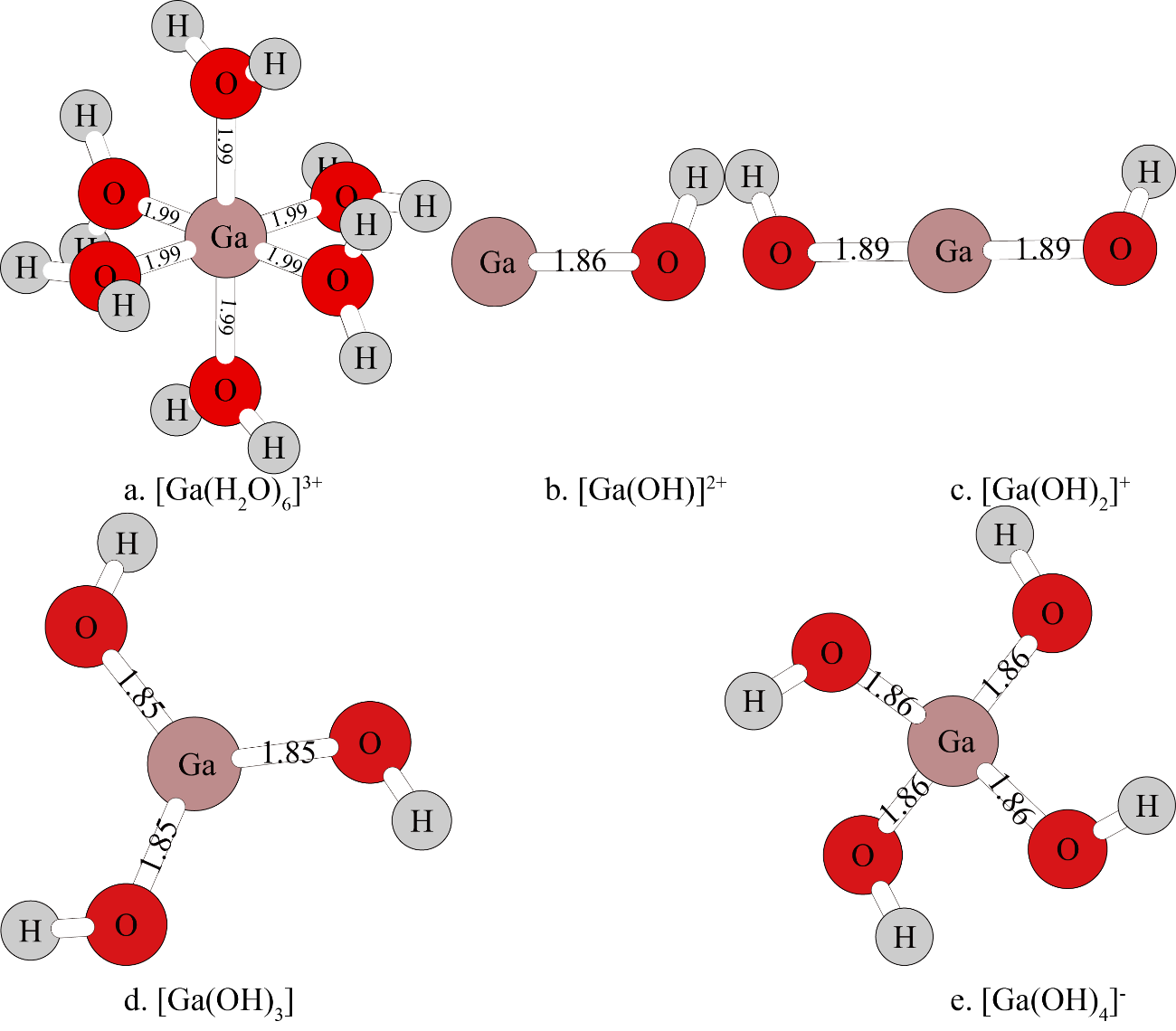


**Figure S3.** Isotopic fractionation data for Ga halides as a function of temperature.


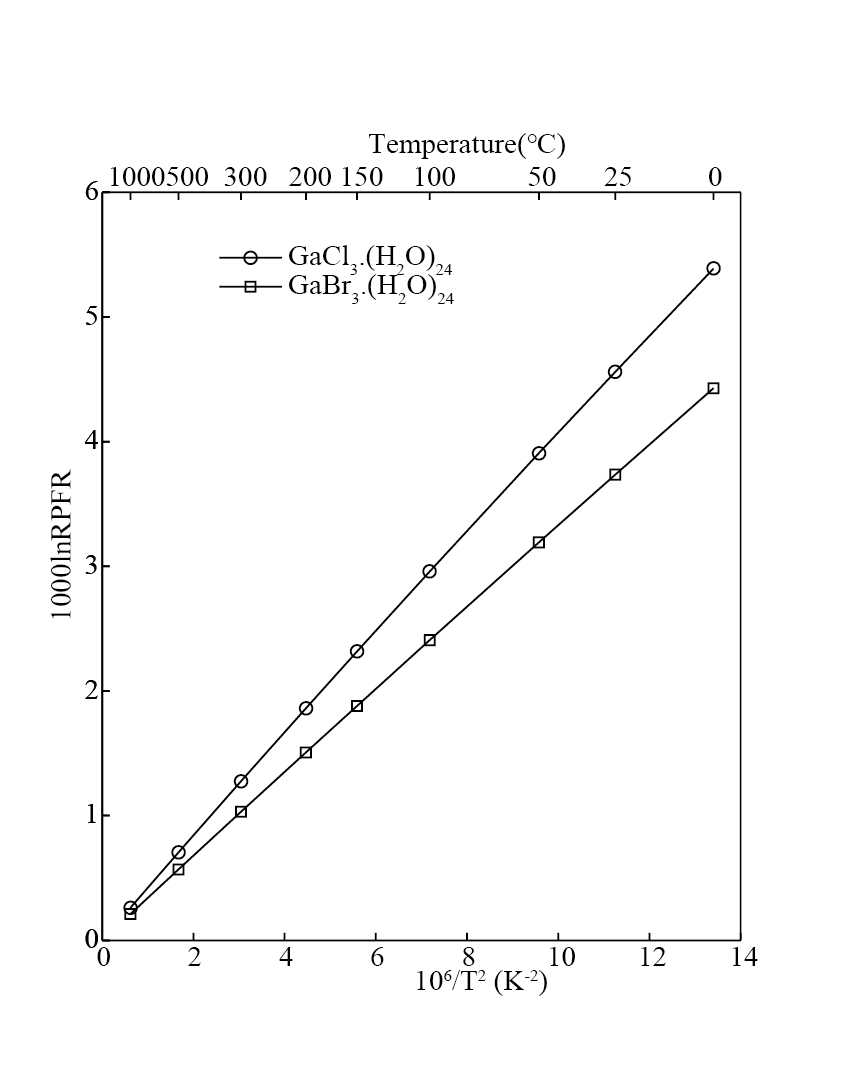


## The details of the calculation method

**the VVCM method**

In this study, the VVCM method were used to simulate solid minerals. Firstly, the crystal structures were obtained for Ga-bearing minerals from the crystal database, and then obtained the mineral fragment structure required for this study. The outermost atoms were fixed in one of the most common ways, by applying virtual charges to the outermost atoms of the mineral fragments. The amount of virtual charges needed to be added to the mineral fragments should be equal to the amount of research systems to ensure the electrical neutrality.

In recent years, the field of theoretical computational geochemistry has yielded an enormous important works. What distinguishes these studies is the method they used. The core problem is how to obtain the simple harmonic vibration frequency of minerals or other matters. In this study, Ga-bearing minerals are simulated by the volume variable cluster model (VVCM) method (a kind of cluster-model based method). With the rapid development of computing power, molecular clusters with dozens, hundreds, or even thousands of atoms can be modeled very well.

The isotope effect is a kind of local property of minerals which has the closest relationship with the central bonds. If the clusters have more than two or three chemical bonds (for atoms of interest, such as Ga), the effect of the external bonds is going to be minimal. Hence, the isotope effect mostly affected by the next nearest neighborhood atoms of the interest atoms (i.e., the NNN rule).

The advantage of the method of VVCM is that the whole mineral fragment could be freely optimized. Under the VVCM treatment, the outermost shell atoms of fragments are removed and all the atoms in VVCM could be freely optimized. This treatment will yield a more reasonable result. In this study, the distance between the virtual charge and the outermost atom is freely adjustable. For a mineral fragment, several structural optimizations with different distances are needed until the structure with the lowest energy is obtained, that is, the most stable structure. A very critical principle is that the same theoretical basis level must be used for structural optimization and frequency calculation.

When constructing the mineral molecular clusters, the atoms of interest must be placed at the center of the mineral fragment. In this study, all the atoms in the center are Ga. After the modeling was done, hundreds of virtual electric charges were placed around the mineral fragments in order to keep the structure electrically neutral. In other words, during the structural optimization and frequency calculation, the structure should be at or close to electric neutrality. The basis set of B3LYP/6-311+G(d) is used for all Ga-bearing minerals of this study.

## Specific details of aqueous solution simulation

In aqueous solution, the major Ga^3+^-bearing species are [Ga(H_2_O)_6_]^3+^, [Ga(OH)]^2+^, [Ga(OH)_2_]^+^, [Ga(OH)_3_] and [Ga(OH)_4_]^-^. Take [Ga(H_2_O)_6_]^3+^ for example, the six H_2_O molecules bonded to the Ga^3+^ were taken as the first-coordination shell of this cluster. The structure of [Ga(H_2_O)_6_]^3+^ has the octahedral structure. Then, the structure optimization and the frequency calculation of the initial structure will be implemented. After that, another six water molecules will be added to the cluster to form [Ga(H_2_O)_6_]^3+^.(H_2_O)_6_. These six water molecules are taken as the second-coordination shell of Ga^3+^. Repeated this process with the same approach until obtained [Ga(H_2_O)_6_]^3+^.(H_2_O)_24_ and do the structure optimization and frequency calculation. When the number of water molecules is equal to 24 and 30, the RPFRs of these complexes tend to the same RPFR value. Hence, the [Ga(H_2_O)_6_]^3+^.(H_2_O)_24_ will be used to represent Ga^3+^-bearing species in solution. For other Ga^3+^-bearing species, the same method will be used. In order to get a more reasonable result, the calculations were repeated for each configuration four times, and then took their average value as the final result. That is, four structures were built with water molecules surrounded in totally four different ways, the average was obtained through arithmetic average. Here, one point that must be emphasized is that when do the geometry optimization and frequency calculation, the same theoretical level will be used. Even each structural optimization and frequency calculation can take a long time under the high theoretical basis set, it is not proper to pre-optimize the geometry with a lower basis set. In this way, an inaccurate local structure will be obtained and this difference could not be eliminated with the improvement of the theoretical level.
